# A procedure for rule extraction from a Self-Organising plasma disruption predictor for JET

**DOI:** 10.1038/s41598-026-38318-9

**Published:** 2026-04-10

**Authors:** Samuele Setzu, Enrico Aymerich, Alessandra Fanni, Fabio Pisano, Giuliana Sias, Barbara Cannas, The JET contributors, C.F. Maggi, D. Abate, N. Abid, P. Abreu, O. Adabonyan, M. Afzal, I. Ahmad, M. Akhtar, R. Albanese, S. Aleiferis, E. Alessi, P. Aleynikov, P. Aleynikov, J. Alguacil, J. Alhage, M. Ali, H. Allen, M. Allinson, M. Alonzo, E. Alves, R. Ambrosino, E.Andersson Sundén, P. Andrew, M. Angelone, C. Angioni, I. Antoniou, L. Appel, C. Appelbee, C. Aramunde, M. Ariola, G. Arnoux, G. Artaserse, J.F. Artaud, W. Arter, V. Artigues, F.J. Artola, A. Ash, O. Asztalos, D. Auld, F. Auriemma, Y. Austin, L. Avotina, J. Ayllón, E. Aymerich, A. Baciero, L. Bähner, F. Bairaktaris, I. Balboa, M. Balden, N. Balshaw, V.K. Bandaru, J. Banks, A.Banon Navarro, C. Barcellona, O. Bardsley, M. Barnes, R. Barnsley, M. Baruzzo, M. Bassan, A. Batista, P. Batistoni, L. Baumane, B. Bauvir, L. Baylor, C. Bearcroft, P. Beaumont, D. Beckett, A. Begolli, M. Beidler, N. Bekris, M. Beldishevski, E. Belli, F. Belli, S. Benkadda, J. Bentley, E. Bernard, J. Bernardo, M. Bernert, M. Berry, L. Bertalot, H. Betar, M. Beurskens, P.G. Bhat, S. Bickerton, J. Bielecki, T. Biewer, R. Bilato, P. Bílková, G. Birkenmeier, R. Bisson, J.P.S. Bizarro, P. Blatchford, A. Bleasdale, V. Bobkov, A. Boboc, A. Bock, G. Bodnar, P. Bohm, L. Bonalumi, N. Bonanomi, D. Bonfiglio, X. Bonnin, P. Bonofiglo, J. Booth, D. Borba, D. Borba, D. Borodin, I. Borodkina, T.O.S.J. Bosman, C. Bourdelle, M. Bowden, I.Boˇzicˇevi´c Mihali´c, S.C. Bradnam, B. Breizman, S. Brezinsek, D. Brida, M. Brix, P. Brown, D. Brunetti, M. Buckley, J. Buermans, H. Bufferand, P. Buratti, A. Burckhart, A. Burgess, A. Buscarino, A. Busse, D. Butcher, G. Calabro, L. Calacci, R. Calado, R. Canavan, B. Cannas, M. Cannon, M. Cappelli, S. Carcangiu, P. Card, A. Cardinali, S. Carli, P. Carman, D. Carnevale, B. Carvalho, I.S. Carvalho, P. Carvalho, I. Casiraghi, F.J. Casson, C. Castaldo, J.P. Catalan, N. Catarino, F. Causa, M. Cavedon, M. Cecconello, L. Ceelen, C.D. Challis, B. Chamberlain, R. Chandra, C.S. Chang, A. Chankin, B. Chapman, P. Chauhan, M. Chernyshova, A. Chiariello, G.-C. Chira, P. Chmielewski, A. Chomiczewska, L. Chone, J. Cieslik, G. Ciraolo, D. Ciric, J. Citrin, Ł. Ciupinski, R. Clarkson, M. Cleverly, P. Coates, V. Coccorese, Coelho, J.W. Coenen, I.H. Coffey, A. Colangeli, L. Colas, J. Collins, Conroy, C. Contré, N.J. Conway, D. Coombs, P. Cooper, S. Cooper, L. Cordaro, C. Corradino, Y. Corre, G. Corrigan, D. Coster, T. Craciunescu, S. Cramp, D. Craven, R. Craven, G. Croci, D. Croft, K. Crombé, T. Cronin, N. Cruz, A. Cufar, A. Cullen, A.Dal Molin, S. Dalley, P. David, A. Davies, J. Davies, S. Davies, G. Davis, K. Dawson, S. Dawson, I. Day, G. De Tommasi, J. Deane, M. Dearing, M. De Bock, J. Decker, R. Dejarnac, E. Delabie, E. de la Cal, E. de la Luna, D. Del Sarto, A. Dempsey, W. Deng, A. Dennett, G.L. Derks, G. De Temmerman, F. Devasagayam, P. de Vries, P. Devynck, A. di Siena, D. Dickinson, T. Dickson, M. Diez, P. Dinca, T. Dittmar, L. Dittrich, J. Dobrashian, T. Dochnal, A.J.H. Donné, W. Dorland, S. Dorling, S. Dormido-Canto, R. Dotse, D. Douai, S. Dowson, R. Doyle, M. Dreval, P. Drews, G. Drummond, Ph. Duckworth, H.G. Dudding, R. Dumont, P. Dumortier, D. Dunai, T. Dunatov, M. Dunne, I. Duran, F. Durodié, R. Dux, T. Eade, E. Eardley, J. Edwards, T. Eich, A. Eksaeva, H. El-Haroun, R.D. Ellis, G. Ellwood, Elsmore, S. Emery, G. Ericsson, B. Eriksson, F. Eriksson, J. Eriksson, L.G. Eriksson, L.G. Eriksson, S. Ertmer, G. Evans, S. Evans, E. Fable, D. Fagan, M. Faitsch, Fajardo Jimenez, M. Falessi, A. Fanni, T. Farmer, I. Farquhar, B. Faugeras, S. Fazini´c, N. Fedorczak, K. Felker, R. Felton, H. Fernandes, D.R. Ferreira, J. Ferreira, G. Ferro, J. Fessey, O. Février, O. Ficker, A.R. Field, A. Figueiredo, J. Figueiredo, A. Fil, N. Fil, P. Finburg, U. Fischer, G. Fishpool, L. Fittill, M. Fitzgerald, D. Flammini, J. Flanagan, S. Foley, N. Fonnesu, M. Fontana, J.M. Fontdecaba, L. Fortuna, E. Fortuna-Zalesna, M. Fortune, C. Fowler, P. Fox, O. Franklin, E. Fransson, L. Frassinetti, R. Fresa, D. Frigione, T. Fülöp, M. Furseman, S. Gabriellini, D. Gadariya, S. Gadgil, K. Gál, S. Galeani, A. Galkowski, D. Gallart, M. Gambrioli, T. Gans, J. Garcia, M. García-Munoz, L. Garzotti, J. Gaspar, R. Gatto, P. Gaudio, D. Gear, T. Gebhart, S. Gee, M. Gelfusa, R. George, S.N. Gerasimov, R. Gerru, G. Gervasini, M. Gethins, Z. Ghani, M. Gherendi, P.I. Gherghina, F. Ghezzi, L. Giacomelli, C. Gibson, L. Gil, M.R. Gilbert, A. Gillgren, E. Giovannozzi, C. Giroud, G. Giruzzi, J. Goff, V. Goloborodko, Gomes, J.-F. Gomez, B. Gonçalves, M. Goniche, J. Gonzalez-Martin, A. Goodyear, Gore, G. Gorini, T. Görler, N. Gotts, E. Gow, J.P. Graves, J. Green, H. Greuner, E. Grigore, F. Griph, W. Gromelski, M. Groth, C. Grove, R. Grove, N. Gupta, S. Hacquin, L. Hägg, A. Hakola, M. Halitovs, J. Hall, C.J. Ham, M. Hamed, M.R. Hardman, Y. Haresawa, G. Harrer, J.R. Harrison, D. Harting, D.R. Hatch, T. Haupt, J. Hawes, N.C. Hawkes, J. Hawkins, S. Hazael, J. Hearmon, P. Heesterman, P. Heinrich, M. Held, W. Helou, O. Hemming, S.S. Henderson, R. Henriques, R.B. Henriques, D. Hepple, J. Herfindal, G. Hermon, J.C. Hillesheim, K. Hizanidis, A. Hjalmarsson, A. Ho, J. Hobirk, O. Hoenen, C. Hogben, A. Hollingsworth, S. Hollis, E. Hollmann, M. Hölzl, M. Hook, M. Hoppe, J. Horácˇek, N. Horsten, N. Horsten, A. Horton, L.D. Horton, L. Horvath, S. Hotchin, Z. Hu, Z. Huang, E. Hubenov, A. Huber, V. Huber, T. Huddleston, G.T.A. Huijsmans, Y. Husain, P. Huynh, A. Hynes, D. Iglesias, M.V. Iliasova, M. Imrísek, J. Ingleby, P. Innocente, V. Ioannou-Sougleridis, N. Isernia, I. Ivanova-Stanik, E. Ivings, S. Jachmich, T. Jackson, A.S. Jacobsen, P. Jacquet, H. Järleblad, A. Järvinen, F. Jaulmes, N. Jayasekera, F. Jenko, I. Jepu, E. Joffrin, T. Johnson, J. Johnston, C. Jones, E. Jones, G. Jones, L. Jones, T.T.C. Jones, A. Joyce, M. Juvonen, A. Kallenbach, P. Kalnina, D. Kalupin, P. Kanth, A. Kantor, A. Kappatou, O. Kardaun, J. Karhunen, E. Karsakos, Ye.O. Kazakov, V. Kazantzidis, D.L. Keeling, W. Kelly, M. Kempenaars, Kennedy, K. Khan, E. Khilkevich, C. Kiefer, H.T. Kim, J. Kim, S.H. Kim, D.B. King, D.J. Kinna, V.G. Kiptily, A. Kirjasuo, K.K. Kirov, A. Kirschner, T. Kiviniemi, G. Kizane, C. Klepper, A. Klix, G. Kneale, M. Knight, P. Knight, R. Knights, S. Knipe, U. Knoche, M. Knolker, M. Kocan, F. Köchl, G. Kocsis, J.T.W. Koenders, Y. Kolesnichenko, Y. Kominis, M. Kong, B. Kool, V. Korovin, S.B. Korsholm, B. Kos, D. Kos, M. Koubiti, Y. Kovtun, E. Kowalska-Strze˛ciwilk, K. Koziol, Y. Krasikov, A. Krasilnikov, V. Krasilnikov, M. Kresina, A. Kreter, K. Krieger, A. Krivska, U. Kruezi, I. Ksia˛zek, H. Kumpulainen, B. Kurzan, S. Kwak, O.J. Kwon, B. Labit, M. Lacquaniti, A. Lagoyannis, L. Laguardia, A. Laing, V. Laksharam, N. Lam, H.T. Lambertz, B. Lane, M. Langley, E.Lascas Neto, E. Łaszynska´, K.D. Lawson, A. Lazaros, E. Lazzaro, G. Learoyd, C. Lee, K. Lee, S. Leerink, T. Leeson, X. Lefebvre, H.J. Leggate, J. Lehmann, M. Lehnen, D. Leichtle, F. Leipold, I. Lengar, M. Lennholm, E.Leon Gutierrez, L.A. Leppin, E. Lerche, A. Lescinskis, S. Lesnoj, L. Lewin, J. Lewis, J. Likonen, Ch. Linsmeier, X. Litaudon, E. Litherland-Smith, F. Liu, T. Loarer, A. Loarte, R. Lobel, B. Lomanowski, P.J. Lomas, J. Lombardo, R. Lorenzini, S. Loreti, V.P. Loschiavo, M. Loughlin, T. Lowe, C. Lowry, T. Luce, R. Lucock, T.Luda Di Cortemiglia, M. Lungaroni, C.P. Lungu, T. Lunt, V. Lutsenko, B. Lyons, J. Macdonald, E. Macusova, R. Mäenpää, C.F. Maggi, H. Maier, J. Mailloux, S. Makarov, P. Manas, A. Manning, P. Mantica, M.J. Mantsinen, J. Manyer, A. Manzanares, Ph. Maquet, M. Maraschek, G. Marceca, G. Marcer, C. Marchetto, O. Marchuk, A. Mariani, G. Mariano, M. Marin, A.Marin Roldan, M. Marinelli, T. Markovicˇ, L. Marot, C. Marren, S. Marsden, S. Marsen, J. Marsh, R. Marshall, L. Martellucci, A.J. Martin, C. Martin, R. Martone, S. Maruyama, M. Maslov, M. Mattei, G.F. Matthews, D. Matveev, E. Matveeva, A. Mauriya, F. Maviglia, M. Mayer, M.L. Mayoral, S. Mazzi, S. Mazzi, C. Mazzotta, R. McAdams, P.J. McCarthy, P. McCullen, R. McDermott, D.C. McDonald, D. McGuckin, V. McKay, L. McNamee, A. McShee, D. Mederick, M. Medland, S. Medley, K. Meghani, A.G. Meigs, S. Meitner, S. Menmuir, K. Mergia, S. Mianowski, P. Middleton, J. Mietelski, K. Mikszuta-Michalik, D. Milanesio, E. Milani, E. Militello-Asp, F. Militello, J. Milnes, A. Milocco, S. Minucci, I. Miron, J. Mitchell, J. Mlynár, J. Mlynár, V. Moiseenko, P. Monaghan, I. Monakhov, A. Montisci, S. Moon, R. Mooney, S. Moradi, R.B. Morales, Morgan, F. Moro, J. Morris, T. Mrowetz, L. Msero, S. Munot, A. Munoz-Perez, Muraglia, A. Murari, A. Muraro, B. N’Konga, Y.S. Na, F. Nabais, R. Naish, F. Napoli, E. Nardon, V. Naulin, M.F.F. Nave, R. Neu, S. Ng, M. Nicassio, D. Nicolai, A.H. Nielsen, S.K. Nielsen, D. Nina, C. Noble, C.R. Nobs, M. Nocente, H. Nordman, S. Nowak, H. Nyström, J. O’Callaghan, M. O’Mullane, C. O’Neill, C. Olde, H.J.C. Oliver, R. Olney, J. Ongena, G.P. Orsitto, A. Osipov, R. Otin, N. Pace, L.W. Packer, E. Pajuste, D. Palade, J. Palgrave, O. Pan, N. Panadero, T. Pandya, E. Panontin, A. Papadopoulos, G. Papadopoulos, G. Papp, V.V. Parail, A. Parsloe, K. Paschalidis, M. Passeri, A. Patel, A. Pau, G. Pautasso, R. Pavlichenko, A. Pavone, E. Pawelec, C. Paz-Soldan, A. Peacock, M. Pearce, I.J. Pearson, E. Peluso, C. Penot, K. Pepperell, A. Perdas, T. Pereira, E.Perelli Cippo, C.Perez von Thun, D. Perry, P. Petersson, G. Petravich, N. Petrella, M. Peyman, L. Pigatto, M. Pillon, S. Pinches, G. Pintsuk, C. Piron, A. Pironti, F. Pisano, R. Pitts, U. Planck, N. Platt, V. Plyusnin, M. Podesta, G. Pokol, F.M. Poli, O.G. Pompilian, M. Poradzinski, M. Porkolab, C. Porosnicu, G. Poulipoulis, A.S. Poulsen, I. Predebon, A. Previti, D. Primetzhofer, G. Provatas, G. Pucella, P. Puglia, K. Purahoo, O. Putignano, T. Pütterich, A. Quercia, G. Radulescu, V. Radulovic, R. Ragona, M. Rainford, P. Raj, M. Rasinski, D. Rasmussen, J. Rasmussen, J.J. Rasmussen, A. Raso, G. Rattá, S. Ratynskaia, R. Rayaprolu, M. Rebai, A. Redl, D. Rees, D. Réfy, Reichle, H. Reimerdes, B.C.G. Reman, C. Reux, S. Reynolds, D. Rigamonti, E. Righi, F.G. Rimini, J. Risner, J.F. Rivero-Rodriguez, C.M. Roach, J. Roberts, R. Robins, Robinson, D. Robson, S. Rode, P. Rodrigues, P. Rodriguez-Fernandez, S. Romanelli, J. Romazanov, E. Rose, C. Rose-Innes, R. Rossi, S. Rowe, D. Rowlands, C. Rowley, M. Rubel, G. Rubinacci, G. Rubino, M. Rud, J. RuizRuiz, F. Ryter, S. Saarelma, A. Sahlberg, M. Salewski, A. Salmi, R. Salmon, F. Salzedas, F. Sanchez, I. Sanders, D. Sandiford, F. Sanni, O. Sauter, P. Sauvan, G. Schettini, A. Shevelev, A.A. Schekochihin, K. Schmid, B.S. Schmidt, S. Schmuck, M. Schneider, P.A. Schneider, N. Schoonheere, R. Schramm, D. Scoon, S. Scully, M. Segato, J. Seidl, L. Senni, J. Seo, G. Sergienko, M. Sertoli, S.E. Sharapov, R. Sharma, A. Shaw, R. Shaw, H. Sheikh, U. Sheikh, N. Shi, P. Shigin, D. Shiraki, G. Sias, M. Siccinio, B. Sieglin, S.A. Silburn, A. Silva, C. Silva, J. Silva, D. Silvagni, D. Simfukwe, J. Simpson, P. Sirén, A. Sirinelli, H. Sjöstrand, N. Skinner, J. Slater, T. Smart, R.D. Smirnov, N. Smith, P. Smith, T. Smith, J. Snell, L. Snoj, E.R. Solano, V. Solokha, C. Sommariva, K. Soni, M. Sos, J. Sousa, C. Sozzi, T. Spelzini, F. Spineanu, L. Spolladore, D. Spong, C. Srinivasan, G. Staebler, A. Stagni, I. Stamatelatos, M.F. Stamp, Z.ˇ Stancarˇ, P.A. Staniec, G. Stankunas, M. Stead, Stein-Lubrano, A. Stephen, J. Stephens, P. Stevenson, C. Steventon, M. Stojanov, D.A. St-Onge, P. Strand, S. Strikwerda, C.I. Stuart, S. Sturgeon, H.J. Sun, S. Surendran, W. Suttrop, J. Svensson, J. Svoboda, R. Sweeney, G. Szepesi, M. Szoke, T. Tadi´c, B. Tal, T. Tala, P. Tamain, K. Tanaka, W. Tang, G. Tardini, M. Tardocchi, D. Taylor, A.S. Teimane, G. Telesca, A. Teplukhina, A. Terra, D. Terranova, N. Terranova, D. Testa, B. Thomas, V.K. Thompson, A. Thorman, A.S. Thrysoe, W. Tierens, R.A. Tinguely, A. Tipton, H. Todd, M. Tomeˇs, A. Tookey, P. Tsavalas, D. Tskhakaya, L.-P. Turic˘a, A. Turner, I. Turner, M. Turner, M.M. Turner, G. Tvalashvili, A. Tykhyy, S. Tyrrell, A. Uccello, V. Udintsev, A. Vadgama, D.F. Valcarcel, A. Valentini, M. Valisa, M. Vallar, M. Valovic, M. Van Berkel, K.L. van de Plassche, M. van Rossem, D. Van Eester, J. Varela, J. Varje, T. Vasilopoulou, G. Vayakis, M. Vecsei, J. Vega, M. Veis, P. Veis, S. Ventre, M. Veranda, G. Verdoolaege, C. Verona, G.Verona Rinati, E. Veshchev, N. Vianello, E. Viezzer, L. Vignitchouk, R. Vila, R. Villari, F. Villone, P. Vincenzi, A. Vitins, Z. Vizvary, M. Vlad, I. Voldiner, U. Von Toussaint, P. Vondrácˇek, B. Wakeling, M. Walker, R. Walker, M. Walsh, R. Walton, E. Wang, F. Warren, R. Warren, J. Waterhouse, C. Watts, T. Webster, M. Weiland, H. Weisen, M. Weiszflog, N. Wendler, A. West, M. Wheatley, S. Whetham, A. Whitehead, D. Whittaker, A. Widdowson, S. Wiesen, M. Willensdorfer, J. Williams, I. Wilson, T. Wilson, M. Wischmeier, A. Withycombe, D. Witts, A. Wojcik-Gargula, E. Wolfrum, R. Wood, R. Woodley, R. Worrall, I. Wyss, T. Xu, D. Yadykin, Y. Yakovenko, Y. Yang, V. Yanovskiy, R. Yi, I. Young, R. Young, B. Zaar, R.J. Zabolockis, L. Zakharov, P. Zanca, A. Zarins, D.Zarzoso Fernandez, K.-D. Zastrow, Y. Zayachuk, M. Zerbini, W. Zhang, B. Zimmermann, M. Zlobinski, A. Zocco, V.K. Zotta, M. Zuin, W. Zwingmann, I. Zychor, WPTE Team, D. Abate, J. Adamek, M. Agostini, C. Albert, F.C.P.Albert Devasagayam, S. Aleiferis, E. Alessi, J. Alhage, S. Allan, J. Allcock, M. Alonzo, Anastasiou, E.Andersson Sunden, C. Angioni, Y. Anquetin, L. Appel, G.M. Apruzzese, M. Ariola, C. Arnas, J.F. Artaud, W. Arter, O. Asztalos, L. Aucone, M.H. Aumeunier, F. Auriemma, J. Ayllon, E. Aymerich, A. Baciero, F. Bagnato, L. Bähner, F. Bairaktaris, P. Balázs, L. Balbinot, I. Balboa, M. Balden, A. Balestri, M.Baquero Ruiz, T. Barberis, C. Barcellona, O. Bardsley, M. Baruzzo, S. Benkadda, T. Bensadon, E. Bernard, M. Bernert, H. Betar, R.Bianchetti Morales, J. Bielecki, R. Bilato, P. Bilkova, W. Bin, G. Birkenmeier, R. Bisson, P. Blanchard, A. Bleasdale, V. Bobkov, A. Boboc, A. Bock, K. Bogar, P. Bohm, T. Bolzonella, F. Bombarda, N. Bonanomi, L. Boncagni, D. Bonfiglio, R. Bonifetto, M. Bonotto, D. Borodin, I. Borodkina, T.O.S.J. Bosman, C. Bourdelle, Bowman, S. Brezinsek, D. Brida, F. Brochard, R. Brunet, Brunetti, V. Bruno, R. Buchholz, J. Buermans, H. Bufferand, P. Buratti, A. Burckhart, J. Cai, R. Calado, J. Caloud, S. Cancelli, F. Cani, B. Cannas, M. Cappelli, S. Carcangiu, A. Cardinali, S. Carli, D. Carnevale, M. Carole, M. Carpita, D. Carralero, F. Caruggi, I.S. Carvalho, I. Casiraghi, A. Casolari, F.J. Casson, C. Castaldo, A. Cathey, F. Causa, J. Cavalier, M. Cavedon, J. Cazabonne, M. Cecconello, L. Ceelen, A. Celora, J. Cerovsky, C.D. Challis, R. Chandra, A. Chankin, B. Chapman, H. Chen, M. Chernyshova, A.G. Chiariello, P. Chmielewski, A. Chomiczewska, C. Cianfarani, G. Ciraolo, J. Citrin, F. Clairet, S. Coda, R. Coelho, J.W. Coenen, I.H. Coffey, C. Colandrea, L. Colas, S. Conroy, C. Contre, N.J. Conway, L. Cordaro, Y. Corre, D. Costa, S. Costea, D. Coster, Courtois, C. Cowley, T. Craciunescu, G. Croci, A.M. Croitoru, K. Crombe, D.J.Cruz Zabala, G. Cseh, T. Czarski, A. Da Ros, A.Dal Molin, M.Dalla Rosa, X. Damizia, O. D’Arcangelo, P. David, M. De Angeli, E. De la Cal, E. De La Luna, G. De Tommasi, J. Decker, R. Dejarnac, D. Del Sarto, G. Derks, C. Desgranges, P. Devynck, S. Di Genova, L.E. di Grazia, A. Di Siena, M. Dicorato, M. Diez, M. Dimitrova, T. Dittmar, L. Dittrich, J.J.Domínguez Palacios Durán, P. Donnel, D. Douai, S. Dowson, S. Doyle, M. Dreval, P. Drews, L. Dubus, R. Dumont, D. Dunai, M. Dunne, A. Durif, F. Durodie, G.Durr Legoupil Nicoud, B. Duval, R. Dux, T. Eich, A. Ekedahl, S. Elmore, G. Ericsson, J. Eriksson, B. Eriksson, F. Eriksson, S. Ertmer, A. Escarguel, B. Esposito, T. Estrada, E. Fable, M. Faitsch, N.Fakhrayi Mofrad, A. Fanni, T. Farley, M. Farník, N. Fedorczak, F. Felici, X. Feng, J. Ferreira, D. Ferreira, N. Ferron, O. Fevrier, O. Ficker, A.R. Field, A. Figueiredo, N. Fil, D. Fiorucci, M. Firdaouss, R. Fischer, M. Fitzgerald, M. Flebbe, M. Fontana, J.Fontdecaba Climent, A. Frank, E. Fransson, L. Frassinetti, D. Frigione, S. Futatani, R. Futtersack, S. Gabriellini, D. Gadariya, D. Galassi, K. Galazka, J. Galdon, S. Galeani, D. Gallart, A. Gallo, C. Galperti, M. Gambrioli, S. Garavaglia, J. Garcia, M.Garcia Munoz, J. Gardarein, L. Garzotti, J. Gaspar, R. Gatto, P. Gaudio, M. Gelfusa, J. Gerardin, S.N. Gerasimov, R.Gerru Miguelanez, G. Gervasini, Z. Ghani, F.M. Ghezzi, G. Ghillardi, L. Giannone, S. Gibson, L. Gil, A. Gillgren, E. Giovannozzi, C. Giroud, G. Giruzzi, T. Gleiter, M. Gobbin, V. Goloborodko, A.González Ganzábal, T. Goodman, V. Gopakumar, G. Gorini, T. Görler, S. Gorno, G. Granucci, D. Greenhouse, G. Grenfell, M. Griener, W. Gromelski, M. Groth, O. Grover, M. Gruca, A. Gude, C. Guillemaut, R. Guirlet, J. Gunn, T. Gyergyek, L. Hagg, A. Hakola, J. Hall, C.J. Ham, M. Hamed, T. Happel, G. Harrer, J. Harrison, D. Harting, N.C. Hawkes, P. Heinrich, S. Henderson, P. Hennequin, R. Henriques, S. Heuraux, J.Hidalgo Salaverri, J. Hillairet, J.C. Hillesheim, A. Hjalmarsson, A. Ho, J. Hobirk, E. Hodille, M. Hölzl, M. Hoppe, J. Horacek, N. Horsten, L. Horvath, M. Houry, K. Hromasova, J. Huang, Z. Huang, A. Huber, E. Huett, P. Huynh, A. Iantchenko, M. Imrisek, P. Innocente, C.Ionita Schrittwieser, H. Isliker, P. Ivanova, I.Ivanova Stanik, M. Jablczynska, S. Jachmich, A.S. Jacobsen, P. Jacquet, A.Jansen van Vuuren, A. Jardin, H. Järleblad, A. Järvinen, F. Jaulmes, T. Jensen, I. Jepu, S. Jessica, E. Joffrin, T. Johnson, A. Juven, J. Kalis, A. Kappatou, J. Karhunen, R. Karimov, A.N. Karpushov, S. Kasilov, Y. Kazakov, P.V. Kazantzidis, D. Keeling, W. Kernbichler, HT. Kim, D.B. King, V.G. Kiptily, A. Kirjasuo, K.K. Kirov, A. Kirschner, A. Kit, T. Kiviniemi, F. Kjær, E. Klinkby, A. Knieps, U. Knoche, M. Kochan, F. Köchl, G. Kocsis, J.T.W. Koenders, L. Kogan, Y. Kolesnichenko, Y. Kominis, M. Komm, M. Kong, B. Kool, S.B. Korsholm, D. Kos, M. Koubiti, J. Kovacic, Y. Kovtun, E.Kowalska Strzeciwilk, K. Koziol, M. Kozulia, A.Krämer Flecken, A. Kreter, K. Krieger, U. Kruezi, O. Krutkin, O. Kudlacek, U. Kumar, H. Kumpulainen, M.H. Kushoro, R. Kwiatkowski, M. La Matina, B. Labit, M. Lacquaniti, L. Laguardia, P. Lainer, P. Lang, M. Larsen, E. Laszynska, K.D. Lawson, A. Lazaros, E. Lazzaro, M.Y.K. Lee, S. Leerink, M. Lehnen, M. Lennholm, E. Lerche, Y. Liang, A. Lier, J. Likonen, O. Linder, B. Lipschultz, A. Listopad, X. Litaudon, E.Litherland Smith, D. Liuzza, T. Loarer, P.J. Lomas, J. Lombardo, N. Lonigro, R. Lorenzini, C. Lowry, T.Luda di Cortemiglia, A.Ludvig Osipov, T. Lunt, V. Lutsenko, E. Macusova, R. Mäenpää, P. Maget, C.F. Maggi, J. Mailloux, S. Makarov, K. Malinowski, P. Manas, A. Mancini, D. Mancini, P. Mantica, M. Mantsinen, J. Manyer, M. Maraschek, Marceca, G. Marcer, C. Marchetto, S. Marchioni, A. Mariani, M. Marin, M. Markl, T. Markovic, D. Marocco, S. Marsden, L. Martellucci, P. Martin, C. Martin, F. Martinelli, L. Martinelli, J.R.Martin Solis, R. Martone, M. Maslov, R. Masocco, M. Mattei, G.F. Matthews, D. Matveev, E. Matveeva, M.L. Mayoral, D. Mazon, S. Mazzi, C. Mazzotta, G. McArdle, R. McDermott, K. McKay, A.G. Meigs, C. Meineri, A. Mele, V. Menkovski, S. Menmuir, A. Merle, H. Meyer, K.Mikszuta Michalik, D. Milanesio, F. Militello, A. Milocco, I.G. Miron, J. Mitchell, R. Mitteau, V. Mitterauer, J. Mlynar, V. Moiseenko, P. Molna, F. Mombelli, C. Monti, A. Montisci, J. Morales, P. Moreau, J.M. Moret, A. Moro, D. Moulton, P. Mulholland, M. Muraglia, A. Murari, A. Muraro, P. Muscente, D. Mykytchuk, F. Nabais, Y. Nakeva, F. Napoli, E. Nardon, M.F. Nave, R.D. Nem, A. Nielsen, S.K. Nielsen, M. Nocente, R. Nouailletas, S. Nowak, H. Nyström, R. Ochoukov, N. Offeddu, S. Olasz, C. Olde, F. Oliva, D. Oliveira, H.J.C. Oliver, P. Ollus, J. Ongena, F.P. Orsitto, N. Osborne, R. Otin, P.Oyola Dominguez, D.I. Palade, S. Palomba, O. Pan, N. Panadero, E. Panontin, A. Papadopoulos, P. Papagiannis, G. Papp, V.V. Parail, C. Pardanaud, J. Parisi, A. Parrott, K. Paschalidis, M. Passoni, F. Pastore, A. Patel, B. Patel, A. Pau, G. Pautasso, R. Pavlichenko, E. Pawelec, B. Pegourie, G. Pelka, E. Peluso, A. Perek, E.Perelli Cippo, C.Perez Von Thun, P. Petersson, G. Petravich, Y. Peysson, V. Piergotti, L. Pigatto, C. Piron, L. Piron, A. Pironti, F. Pisano, U. Plank, B. Ploeckl, V. Plyusnin, A. Podolnik, Y. Poels, G. Pokol, J. Poley, G. Por, M. Poradzinski, F. Porcelli, L. Porte, C. Possieri, A. Poulsen, I. Predebon, G. Pucella, M. Pueschel, P. Puglia, O. Putignano, T. Pütterich, V. Quadri, A. Quercia, M. Rabinski, L. Radovanovic, R. Ragona, H. Raj, M. Rasinski, J. Rasmussen, G. Ratta, S. Ratynskaia, R. Rayaprolu, M. Rebai, A. Redl, D. Rees, D. Refy, M. Reich, H. Reimerdes, B.C.G. Reman, O. Renders, C. Reux, D. Ricci, M. Richou, S. Rienacker, D. Rigamonti, F. Rigollet, F.G. Rimini, D. Ripamonti, N. Rispoli, N. Rivals, J.F.Rivero Rodriguez, C. Roach, G. Rocchi, S. Rode, P. Rodrigues, J. Romazanov, C.F.Romero Madrid, J. Rosato, R. Rossi, G. Rubino, J. RuedaRueda, J. RuizRuiz, P. Ryan, D. Ryan, S. Saarelma, R. Sabot, M. Salewski, A. Salmi, L. Sanchis, A. Sand, J. Santos, K. Särkimäki, M. Sassano, O. Sauter, G. Schettini, S. Schmuck, P. Schneider, N. Schoonheere, R. Schramm, R. Schrittwieser, C. Schuster, N. Schwarz, F. Sciortino, M.Scotto DAbusco, S. Scully, A. Selce, L. Senni, M. Senstius, G. Sergienko, S.E. Sharapov, R. Sharma, A. Shaw, U. Sheikh, G. Sias, B. Sieglin, S.A. Silburn, C. Silva, A. Silva, D. Silvagni, B.Simmendefeldt Schmidt, L. Simons, J. Simpson, L. Singh, S. Sipilä, Y. Siusko, S. Smith, A. Snicker, E.R. Solano, V. Solokha, M. Sos, C. Sozzi, F. Spineanu, G. Spizzo, M. Spolaore, L. Spolladore, C. Srinivasan, A. Stagni, Z. Stancar, G. Stankunas, J. Stober, P. Strand, C.I. Stuart, F. Subba, G.Y. Sun, H.J. Sun, W. Suttrop, J. Svoboda, T. Szepesi, G. Szepesi, B. Tal, T. Tala, P. Tamain, G. Tardini, M. Tardocchi, D. Taylor, G. Telesca, A. Tenaglia, A. Terra, D. Terranova, D. Testa, C. Theiler, E. Tholerus, B. Thomas, E. Thoren, A. Thornton, A. Thrysoe, Q. TICHIT, W. Tierens, A. Titarenko, P. Tolias, E. Tomasina, M. Tomes, E. Tonello, A. Tookey, M.Toscano Jiménez, C. Tsironis, E. Tsitrone, E. Tsitrone, C. Tsui, A. Tykhyy, M. Ugoletti, M. Usoltseva, D.F. Valcarcel, A. Valentini, M. Valisa, M. Vallar, M. Valovic, SI. Valvis, M. van Berkel, D. Van Eester, S. Van Mulders, M. van Rossem, R. Vann, B. Vanovac, J.Varela Rodriguez, J. Varje, S. Vartanian, M. Vecsei, L.Velarde Gallardo, M. Veranda, T. Verdier, G. Verdoolaege, K. Verhaegh, L. Vermare, G.Verona Rinati, N. Vianello, J. Vicente, E. Viezzer, L. Vignitchouk, F. Villone, B. Vincent, P. Vincenzi, M.O. Vlad, G. Vogel, I. Voitsekhovitch, I. Voldiner, P. Vondracek, N.M.T. VU, T. Vuoriheimo, C. Wade, E. Wang, T. Wauters, M. Weiland, H. Weisen, N. Wendler, D. Weston, A. Widdowson, S. Wiesen, M. Wiesenberger, T. Wijkamp, M. Willensdorfer, T. Wilson, M. Wischmeier, A. Wojenski, C. Wuethrich, I. Wyss, L. Xiang, S. Xu, D. Yadykin, Y. Yakovenko, H. Yang, V. Yanovskiy, R. Yi, B. Zaar, G. Zadvitskiy, L. Zakharov, P. Zanca, D. Zarzoso, Y. Zayachuk, J. Zebrowski, M. Zerbini, P. Zestanakis, C.F.B. Zimmermann, M. Zlobinski, A. Zohar, V.K. Zotta, X. Zou, M. Zuin, M. Zurita, I. Zychor

**Affiliations:** 1 Department of Electrical and Electronic Engineering, University of Cagliari, Cagliari, Italy; 2 United Kingdom Atomic Energy Authority, Culham Campus, Abingdon, OX14 3DB Oxfordshire United Kingdom of Great Britain and Northern Ireland; 3 Consorzio RFX (CNR, ENEA, INFN, Universit’a di Padova, Acciaierie Venete SpA), C.so Stati Uniti 4, 35127 Padova, Italy; 4 Instituto de Plasmas e Fus~ao Nuclear, Instituto Superior Técnico, Universidade de Lisboa, 1049-001 Lisboa, Portugal; 5 Consorzio CREATE, Via Claudio 21, 80125 Napoli, Italy; 6 Institute for Plasma Science and Technology, CNR, via R. Cozzi 53, 20125 Milano, Italy; 7 ITER Organization, Route de Vinon-sur-Verdon, CS 90 046, 13067 Saint Paul Lez Durance Cedex, France; 8 Max-Planck-Institut für Plasmaphysik, D-85748 Garching, Germany; 9 Dept Ingn Energet, Universidad Nacional de Educacion a Distancia, Calle Juan del Rosal 12, E-28040 Madrid, Spain; 10 Department of Applied Physics, Ghent University, 9000 Ghent, Belgium; 11 Dip.to Fusione e Tecnologie per la Sicurezza Nucleare, ENEA C. R. Frascati, via E. Fermi 45, 00044 Frascati (Roma), Italy; 12 Department of Physics and Astronomy, Uppsala University, SE-75120 Uppsala, Sweden; 13 CEA, IRFM, F-13108 Saint Paul Lez Durance, France; 14 Centre for Energy Research, POB 49, H-1525 Budapest, Hungary; 15 University of Latvia, 19 Raina Blvd., Riga, 1586 LV Latvia; 16 Universidad de Sevilla, Sevilla, Spain; 17 Laboratorio Nacional de Fusión, CIEMAT, Madrid, Spain; 18 Fusion Plasma Physics, EECS, KTH Royal Institute of Technology, SE-10044 Stockholm, Sweden; 19 National Technical University of Athens, Iroon Politechniou 9, 157 73 Zografou, Athens, Greece; 20 Dipartimento di Ingegneria Elettrica Elettronica e Informatica, Universit’a degli Studi di Catania, 95125 Catania, Italy; 21 Rudolf Peierls Centre for Theoretical Physics, University of Oxford, Oxford, OX1 3PU United Kingdom of Great Britain and Northern Ireland; 22 Oak Ridge National Laboratory, Oak Ridge, TN 37831 USA; 23 Karlsruhe Institute of Technology, PO Box 3640, D-76021 Karlsruhe, Germany; 24 General Atomics, PO Box 85608, San Diego, CA 92186-5608 USA; 25 Aix-Marseille University, CNRS, PIIM, UMR 7345, 13013 Marseille, France; 26 Universite´de Lorraine, CNRS, F-54000 Nancy, IJL France; 27 Faculty of Mathematics, Department of Experimental Physics, Physics and Informatics, Comenius University, Mlynska dolina F2, 84248 Bratislava, Slovakia; 28 Institute of Nuclear Physics Polish Academy of Sciences (IFJ PAN), Radzikowskiego 152, 31-342 Kraków, Poland; 29 Institute of Plasma Physics of the CAS, Za Slovankou 1782/3, 182 00 Praha 8, Czech Republic; 30 University of Milano-Bicocca, Piazza della Scienza 3, 20126 Milano, Italy; 31 Princeton Plasma Physics Laboratory, James Forrestal Campus, Princeton, NJ 08543 USA; 32 EUROfusion Programme Management Unit, Culham Science Centre, Culham, OX14 3DB United Kingdom of Great Britain and Northern Ireland; 33 Forschungszentrum Jülich GmbH, Institut für Energie- und Klimaforschung, Plasmaphysik, 52425 Jülich, Germany; 34 FOM Institute DIFFER, Eindhoven, Netherlands; 35 Rud¯er Boˇskovi´c Institute, Bijenicˇka 54, 10000 Zagreb, Croatia; 36 Institute for Fusion Studies, University of Texas at Austin, Austin, TX 78712 USA; 37 HeinrichHeine-Universität Düsseldorf, 40225 Düsseldorf, Germany; 38 Laboratory for Plasma Physics LPP-ERM/KMS, B-1000 Brussels, Belgium; 39 Universit’a di Roma Tor Vergata, Via del Politecnico 1, Roma, Italy; 40 University of Tuscia, DEIM, Via del Paradiso 47, 01100 Viterbo, Italy; 41 Istituto dei Sistemi Complessi—CNR and Dipartimento di Energia—Politecnico di Torino, C.so Duca degli Abruzzi 24, 10129 Torino, Italy; 42 Toegepaste Mechanica en Energieconversie, Katholieke Universiteit of Leuven, 3001 Leuven, Belgium; 43 Aalto University, PO Box 14100, FIN-00076 Aalto, Finland; 44 Institute of Plasma Physics and Laser Microfusion, Hery 23, 01-497 Warsaw, Poland; 45 Warsaw University of Technology, Nowowiejska 15/19, 00-665 Warsaw, Poland; 46 Astrophysics Research Centre, School of Mathematics and Physics, Queen’s University, Belfast, BT7 1NN United Kingdom of Great Britain and Northern Ireland; 47 Swiss Plasma Center (SPC), Ecole Polytechnique Fédérale de Lausanne (EPFL), CH-1015 Lausanne, Switzerland; 48 The National Institute for Laser, Plasma and Radiation Physics, Magurele-Bucharest, Romania; 49 Slovenian Fusion Association (SFA), Jozef Stefan Institute, Jamova 39, SI-1000 Ljubljana, Slovenia; 50 Dublin City University (DCU), Dublin, Ireland; 51 Department of Physics, York Plasma Institute, University of York, Heslington, YO10 5DD York United Kingdom of Great Britain and Northern Ireland; 52 EUROfusion Programme Management Unit, Boltzmannstr. 2, 85748 Garching, Germany; 53 Dpto. Informática y Automática, UNED, Madrid, Spain; 54 National Science Center ‘Kharkov Institute of Physics and Technology’, Akademichna 1, Kharkiv, 61108 Ukraine; 55 Department of Space, Earth and Environment, Chalmers University of Technology, SE-41296 Gothenburg, Sweden; 56 European Commission, B-1049 Brussels, Belgium; 57 Université Cote d’Azur, CNRS, LJAD, Parc Valrose, Nice, Inria France; 58 Argonne National Laboratory, Lemont, IL 60439 USA; 59 Department of Physics, Chalmers University of Technology, SE-41296 Gothenburg, Sweden; 60 Dipartimento di Ingegneria Astronautica, Elettrica ed Energetica, SAPIENZA Universit’a di Roma, Via Eudossiana 18, 00184 Roma, Italy; 61 Barcelona Supercomputing Center, Barcelona, Spain; 62 UMR 7343, Aix-Marseille University, CNRS, IUSTI, 13013 Marseille, France; 63 Department of Physics, Technical University of Denmark, Bldg 309, DK-2800 Kgs Lyngby, Denmark; 64 Institute for Nuclear Research, Prospekt Nauky 47, Kyiv, 03680 Ukraine; 65 Harvard University, Harvard Square, Cambridge, MA 02138 USA; 66 University of California, Irvine, CA 92697 USA; 67 VTT Technical Research Centre of Finland, PO Box 1000, FIN-02044 VTT Espoo, Finland; 68 Fusion@ÖAW Österreichische Akademie der Wissenschaften (ÖAW), Technische Universität Wien, Wien, Austria; 69 Eindhoven University of Technology, Eindhoven, Netherlands; 70 University of California at San Diego, La Jolla, CA 92093 USA; 71 Ioffe Physico-Technical Institute, 26 Politekhnicheskaya, St Petersburg, 194021 Russian Federation; 72 NCSR ‘Demokritos’, 153 10, Agia Paraskevi, Attikis Greece; 73 Department of Applied Mathematics and Computer Science, Technical University of Denmark, Bldg 309, DK-2800 Kgs Lyngby, Denmark; 74 Korea Institute of Fusion Energy, Daejeon, Korea; 75 National Centre for Nuclear Research (NCBJ), 05-400 Otwock-Swierk, Poland; 76 Institution ‘Project Center ITER’, Moscow, 123182 Russian Federation; 77 Jülich GmbH, Institut für Energie- und Klimaforschung, Plasmaphysik, 52425 Jülich, Germany; 78 Institute of Physics, Opole University, Oleska 48, 45-052 Opole, Poland; 79 Daegu University, Jillyang, Gyeongsan, 712-174 Gyeongbuk Korea; 80 Department of Nuclear Engineering, Seoul National University, Seoul, Korea; 81 ICREA and Barcelona Supercomputing Center, Barcelona, Spain; 82 Universidad Complutense de Madrid, Madrid, Spain; 83 Department of Physics, University of Basel, Basel, Switzerland; 84 University College Cork (UCC), Cork, Ireland; 85 Politecnico di Torino, Corso Duca degli Abruzzi 24, I-10129 Torino, Italy; 86 Faculty of Nuclear Sciences and Physical Engineering, Czech Technical University in Prague, Bˇrehová 78/7, 115 19 Praha 1, Czech Republic; 87 Space and Plasma Physics, EECS, SE-100 44 Stockholm, KTH Sweden; 88 Columbia University, New York, NY 10027 USA; 89 Consorzio RFX, Corso Stati Uniti 4, 35127 Padova, Italy; 90 Plasma Science and Fusion Center, Massachusetts Institute of Technology, Cambridge, MA 02139 USA; 91 University of Ioannina, Panepistimioupoli Ioanninon, PO Box 1186, 45110 Ioannina, Greece; 92 Faculdade de Engenharia, Universidade do Porto, 4200465 Porto, Portugal; 93 University Roma Tre, via Vito Volterra N◦62, 00146 Rome, CAP Italy; 94 University of Helsinki, PO Box 43, FI-00014 Helsinki, Finland; 95 Lithuanian Energy Institute, Breslaujos g. 3, LT-44403 Kaunas, Lithuania; 96 National Institute for Fusion Science, Oroshi, Toki, Gifu 5095292 Japan; 97 Universidad Carlos III de Madrid, 28911 Leganes, Madrid Spain; 98 V.N. Karazin Kharkiv National University, Kharkiv, Ukraine; 99 CRF—University of Padova, 35127 Padova, Italy; 100 Dipartimento di Fisica ‘G. Galilei’, Universita’ degli Studi di Padova, Padova, Italy; 101 IRFM-CEA Centre de Cadarache, 13108 Sant-Paul-lez-Durance, France; 102 Max Planck Institute for Plasma Physics, Boltzmannstrasse 2, 85748 Garching bei München, Germany; 103 Istituto per la Scienza e la Tecnologia dei Plasmi, CNR, Padova, Italy; 104 Graz University of Technology, Petersgasse 16, 8010 Graz, Austria; 105 Aix Marseille University, CNRS, F-13397 Marseille CEDEX 20, PIIM France; 106 Electromagnetic Engineering and Fusion Science, EECS, KTH Royal Institute of Technology, Stockholm as indicated, SE-10044 Stockholm, Sweden; 107 Institut Jean Lamour, UMR 7198, CNRS-Université de Lorraine, 54500 Vandoeuvrelès-Nancy, France; 108 DIFFER-Dutch Institute for Fundamental Energy Research, Eindhoven, Netherlands; 109 Institut Jean Lamour, Université de Lorraine, Nancy, France; 110 DEIM Department, Universitá degli Studi della Tuscia, Viterbo, Italy; 111 Department of Mechanical Engineering, KU Leuven, Leuven, Belgium; 112 Joˇzef Stefan Institute, Ljubljana, Slovenia–University of Ljubljana, Lyubljana, Slovenia; 113 Aix-Marseille University, CNRS, M2P2 Marseille, France; 114 Department of Physics, Durham University, Durham, DH1 3LE United Kingdom of Great Britain and Northern Ireland; 115 Technische Universität Wien, Wiedner Hauptstr. 8-10/134, A-1040 Wien, Austria; 116 Laboratoire de Physique des Plasmas, Ecole Polytechnique, Palaiseau, France; 117 Institute of Ion Physics and Applied Physics, University of Innsbruck, Innsbruck, Austria; 118 Institute of Electronics, Bulgarian Academy of Sciences (BAS), 72 Tsarigradsko Chaussee, 1784 Sofia, Bulgaria; 119 Loughborough University, Loughborough, Leicestershire United Kingdom of Great Britain and Northern Ireland; 120 Section of Astrophysics, Astronomy and Mechanics, Physics Department, Aristotle University, 541 24 Thessaloniki, GR Greece; 121 Politecnico di Milano, Milan, Italy; 122 Aix Marseille University, CNRS, IUSTI UMR 7343, F-13013 Marseille, France; 123 Laboratory of Nuclear Installation Safety, Lithuanian Energy Institute, Breslaujos Str. 3, LT-44403 Kaunas, Lithuania; 124 Center for Energy Research (CER), University of California-San Diego (UCSD), La Jolla, CA USA; 125 Faculty of Mathematics and Natural Sciences, Heinrich Heine University Düsseldorf, 40225 Düsseldorf, Germany

**Keywords:** Electrical and electronic engineering, Magnetically confined plasmas, Scientific data, Statistics

## Abstract

In a previous paper, a Self-Organizing Map had proven to be able to identify the regions of the plasma operative space characterizing the pre-disruptive phase at JET without relying on any a priori information. One of the strengths of this disruption predictor lies in its inherent self-organization capability. The Self-Organizing Map discovers non-trivial relationships and captures the complicated interplay of device diagnostics on the internal plasma states directly from the experimental data. Moreover, the provided model allows the visualization of high-dimensional plasma parameters and facilitates easy interrogation of the model to understand the reasons behind its correlations. In this paper, an additional step is taken towards the interpretability of models for predicting disruptions by training a Decision Tree to classify the plasma states according to the interpretation provided by the Self-Organizing Map (stable or at high risk of disruptions). The Decision tree provides a set of rules which describe the transition of the plasma towards the pre-disruptive phase as visualized in the Self-Organizing Map. The obtained rules for the database explored in the study identify four regions in the map, two of which are at risk of disruption. These regions correspond to partitions of a 3D space based on the peaking factors of the core and divertor radiation, as well as the Locked Mode. The agreement between the Self-Organizing Map answers and the rules supplied by the Decision Tree is confirmed by the comparison of the performance exhibited by the two models in the prediction of disruptions.

## Introduction

A plasma disruption is a sudden, critical event in a tokamak that results in the loss of plasma confinement and stability. Disruptions often cause a variety of damaging consequences due to localized overheating, excessive stress on machine walls, or even damage to critical components like the first-wall materials or superconducting magnets. Reliable disruption prediction and mitigation systems are considered unavoidable during International Thermonuclear Experimental Reactor (ITER) operations and in the view of the next fusion reactors such as the DEMOnstration Power Plant (DEMO).

There is not a comprehensive theoretical model that describes all types of disruptions. For this reason, data-based models utilizing machine learning (ML) and Neural Networks (NNs) are common approaches for classifying and predicting disruptions. There is a wide body of literature on supervised ML models, which require labeled training data. The performance of the models has improved over the years both for a more appropriate choice of diagnostic signals and input features and for the availability of increasingly powerful data-driven modelling techniques. The most recent approaches, including Deep Learning, Reinforcement Learning (RL), Ensemble methods and Transfer learning leverage advanced algorithms and large-scale data from plasma diagnostics.

Deep NN, including Convolutional Neural Networks (CNNs), Recurrent NNs and Autoencoders, were applied to analyze time-series data and images^1–3^. Examples include a deep learning–based cross-device predictor trained on data from both DIII-D and JET^1^, a deep learning-based real-time disruption predictor implemented in the plasma control system of HL-2A^2^ and a CNN processing plasma profiles for disruption prediction at JET^3^, then improved adding the MHD spectrogram^4^. Furthermore, a deep learning-disruption predictor for KSTAR analyses In-vessel Visible Inspection System video and 0D parameters^5^, while a cross-tokamak predictor for EAST and J-TEXT exploits Convolutional Autoencoders^6^ and adaptive deployment methods based on anomaly detection.

Also, RL was explored for optimizing real-time disruption prediction systems. By continuously learning from the system’s responses, RL can improve its predictive capability, even in dynamic and uncertain environments, by receiving feedback from past predictions and actions. RL has been successfully applied to develop a dynamic model that estimates the likelihood of future tearing instability in DIII-D^7^.

To enhance prediction reliability, ensemble techniques that combine multiple ML models have been widely adopted. Methods like Random Forests (RFs) or gradient boosting allow for better generalization and reduce the likelihood of false positives or negatives in disruption prediction. A real-time disruption predictor using RFS was developed for high-density disruptions and integrated into the plasma control system of EAST tokamak^8^. A data-driven disruption prediction model using the RFs for KSTAR is developed^9^.

Transfer learning, which is the process of adapting a model trained on one dataset for use on another, is gaining attention in disruption prediction. It is particularly valuable in scenarios with limited labelled data, as it enables the transfer of knowledge from different devices or experimental conditions. An explorative data analysis study on C-Mod disruptions database using t-distributed Stochastic Neighbour Embedding (t-SNE)^10^, a dimensionality reduction technique, demonstrated that time sequence data can better separate the disruptive and non-disruptive behaviour compared to the instantaneous plasma state data. The authors designed a multi-machine Hybrid Deep learning disruption predictor that achieves high predictive accuracy on C-Mod, DIII-D and EAST tokamaks with limited hyperparameter tuning. Transfer learning-based methods to predict disruptions trained with J-TEXT data have been successfully applied to port the predictors to EAST^6,11^.

In all these applications, the manual labelling of the training data of Disruption Terminated Experiments (DTEs), to identify when disruption precursors appear, was a heavy and challenging task. To overcome this labelling phase, the authors of the present paper proposed a statistical algorithm capable of identifying a different pre-disruptive phase for each disrupted discharge^12^. Previously, the same authors developed disruption predictors based on anomaly detection which only required samples from Regularly Terminated Experiments (RTEs) labelling them as ‘normal’^13^. Then, a Self-Organising Map disruption predictor is presented. The predictor is a fully unsupervised two-dimensional mapping of the high-dimensional JET operational space. Its primary strength lies in its inherent self-organization capability. Diverging from supervised disruption predictors and earlier approaches suggested by the same authors^12^, it eliminates the need for labelling data of DTEs during training. The Self-Organizing Map, operating without any a priori information, efficiently identifies the regions characterizing the pre-disruptive phase. In other works, precursor onset times using different anomaly detection algorithms are estimated for J-TEXT and EAST^14^.

The ongoing challenge lies in implementing models that can be easily interrogated to understand the reasoning behind their predictions, ensuring a closer connection to underlying physical mechanisms. In the future, interpretable models applied across devices could be a valuable help for shared rule extraction and identification of common patterns towards a more confident extrapolation to ITER.

With manifold mapping, the obtained 2D maps allow one to visualize the high-dimensional plasma parameter space as a 2D projection^15,16^. The component planes make it possible to observe the distribution of individual plasma state parameters on the map. A similar approach used t-SNE to visualize the data representation in a 3D latent space^5^. Instead, other authors propose methods to evaluate the real-time importance of each input signal^2^. Given an existing predictor^1^, researchers introduced a real-time “sensitivity score” to indicate the underlying reasons for the imminent disruption^17^. Other approaches measured the feature importance of 0D parameters in a Transformer-based disruption predictor applying the permutation feature importance algorithm^5^. To enhance interpretability, physics and data-driven methodologies such as fully connected NNs are combined to identify the main macroscopic precursors of disruptions^18^: magnetic instabilities, abnormal kinetic profiles, and radiation patterns. In this paper, a procedure to implement an unsupervised disruption predictor at JET and uncover the rules underlying its behaviour is described. The predictor represents an enhanced version of the SOM^15^; a Decision tree is then employed to reveal the rules governing the operation of the SOM. The present work gives a more in-depth interpretability of the SOM predictor at JET, showing a first rule extraction approach that will be applied also to other machines in future work. The paper is organized as follows: in Section "Decision trees" the Decision Trees are outlined. Section "Disruption prediction through SOM" recalls the SOM proposed for disruption prediction at JET^15^, the database and the performance indexes. An improved version of the SOM predictor is described in Section "SOM analysis" while in Sect. "Rule extraction through decision trees" the rule extraction mechanism is introduced. The adaptability of the rules to the SOM is presented in Sect. "Integrating SOM and decision tree for rule extraction". In Sections "Interpretation of the DTr rules" and "3D plasma parameter space", the extracted rules set are analysed, then a graphical interpretation is given, enhancing the strength of the proposed method. In Section "Conclusions", the conclusions are drawn.

## Decision trees

Decision Trees (DTr) are supervised machine learning algorithms^19^ primarily used for classification tasks. They work by subdividing the input space into subspaces based on the values of input features, each split considering only one feature at a time. This occurs at each node in the tree, where the objective is to separate the data in a way that maximizes the purity or homogeneity of the resulting subsets.

At each node, the algorithm selects the feature and the threshold value (for continuous features) that best splits the data. Each node thus represents a decision on an input feature, and each branch corresponds to a possible outcome. The leaves provide the required classification. The paths from the root to the leaves form the classification rules.

To determine the best feature and corresponding threshold for splitting data at each node, several metrics can be used. In this paper, the Gini impurity index is used as a measure of the degree of impurity within the dataset^19^. The training algorithm evaluates all possible features and corresponding thresholds to find the one that minimizes the impurity index, ultimately aiming to create the purest possible nodes, where the data points are as similar as possible in terms of the target variable.

The tree is built by repeatedly splitting the data based on feature attributes, growing until a stopping criterion is met, such as when all leaves are pure (i.e., belonging to the same class) or when the minimum number of samples per node or maximum tree depth is reached.

## Disruption prediction through SOM

### Database

The data for this study contains experiments from JET campaigns from 2011 to 2020. The database covers earlier campaigns with the ILW until more recent higher power experiments. It has been grouped into three data sets^15^:

1. Campaigns 2011–2013: 127 DTEs and 115 RTEs
2. Campaigns 2016: 29 DTEs and 41 RTEs
3. Campaigns 2019–2020: 37 DTEs and 63 RTEs

In total, the database consists of 193 DTEs and 219 RTEs having a flat-top plasma current higher than 1.5 MA, and a flat-top length greater than 200 $ms$. The flat-top starting time is assumed as the first time instant where the plasma is in X-point configuration. The diagnostic and synthetic signals derived from 1D plasma profiles^16,20^ were:

- Peaking Factor of Electron Temperature (Te_pf_) and Electron Density (Ne_pf_) from HRTS
- Peaking Factor of the Radiation excluding the X point/divertor region (Rad_pf-CVA_) and excluding the core region (Rad_pf-XDIV_), from the Bolometer Horizontal Camera
- Internal Inductance (${l}_{i}$) from the Magnetic equilibrium
- Normalized Locked Mode Amplitude (LM) from the Saddle loops.

Dataset3, related to experiments for baseline scenario studies, suitable for sustained high D–T fusion power, is characterized by higher currents, density, and input power, also exceeding the range of the other two datasets^21^. The dataset consists of all samples of the whole flat top phase of the plasma current; samples from the ramp down one are not taken into account.

The training set consists of 85 DTEs and 70 RTEs from 2011–2013 campaigns and the test set consists of 108 DTEs and 149 RTEs from 2011–2020 campaigns. In total, the training set consists of 179 601 samples, while the test set consists of 901 535 samples.

The signals are sampled every 2 $ms$. In the training set, they have been under sampled to 10 $ms$, except for the final second of the DTEs, to limit map dimensions. The validation of the SOM is carried out by feeding it with the same experiments used for training but with a sampling time of 2 $ms$. While this does not constitute a fully independent evaluation and may underestimate the validation error, it can help reduce overfitting in distinguishing between safe and disruptive regions. The performance of the SOM as disruption predictor is assessed using the independent test set which encompasses experiments from the same campaigns of training set and from subsequent campaigns.

### Performance indexes

At JET, the time required for the Massive Gas Injection system to mitigate the discharge in case of alarm is 10 $ms$. Thus, 10 $ms$ is the minimum warning time, i.e., the minimum time interval between the alarm and the disruption. If the predictor triggers an alarm with a warning time equal to or smaller than 10 $ms$, it is assumed as Tardy Detection (TD). A Missed Alarm (MA) occurs if the disruption predictor does not trigger any alarm. A FA is an alarm triggered by the predictor during an RTE.

In the disruption prediction literature, a more informative figure of merit is defined by the accumulated fraction of detected disruptions as a function of the warning time. It allows us to read, in a unique graph, besides the successful prediction and the tardy detections, also a general overview of the premature detections and the alarm anticipation times.

### The SOM predictor

SOM is an unsupervised NN, generally used for clustering. The SOM can map an N-dimensional input space in a low dimensional output space, preserving the topology of input data^22^. So, similar data in the input space will be associated with the same cluster in the output space. The choice of the SOM is motivated by several factors, including its self-organizing capability and the ability to visualize data in a low-dimensional space, which enhances the model’s interpretability.

In the lower dimensional output space then, every cluster represents a vector with the same dimensionality as the input space. After the model is trained, every sample can be associated with the nearest cluster in terms of Euclidean distance, called Best Matching Unit (BMU).

A SOM disruption predictor, SOM2^15^, has been trained and validated using the same 85 DTEs and 70 RTEs from Dataset1 as in previous work^23^. Dataset1 consists of 6 diagnostic signals. The remaining 108 DTEs and 149 RTEs have been used for testing the model’s performance. The developed model can find by itself the cluster labels, i.e., it can separate the safe clusters and the disruptive ones.

Testing the SOM performance in terms of disruption prediction, many FAs were triggered in the frontier between safe and disruptive region; thus, a “safe” class label was assigned to every frontier cluster. To identify novel samples, for each BMU, the minimum and maximum values of each variable across the training samples associated to the BMU itself are evaluated. For a new sample, if the value of any variable exceeds the maximum by more than 10% or falls below the minimum by more than 10%, the sample is labelled as novel. In this case the eventual alarm was not triggered^15^.

## SOM analysis

In this paper, the SOM predictor was retrained using the same experiments, and the same training, validation and test sets previously employed by the authors^15^. However, the Radiation peaking factor calculation was modified with a causal preprocessing for cleaning bolometer noisy channels. For each experiment and for each of the 20 channels in the first 200 $ms$ of the flat top phase, the trend of the signal is removed by subtracting the signal with the moving average. To identify channels with corrupted and out of range values, the standard deviation of each radiation signal is computed and a threshold *Th* on standard deviations is determined:

1 $$Th= \mu \left({\widehat{\sigma }}_{1}, {\widehat{\sigma }}_{2},\dots ,{\widehat{\sigma }}_{20}\right)+3\sigma \left({\widehat{\sigma }}_{1}, {\widehat{\sigma }}_{2},\dots ,{\widehat{\sigma }}_{20}\right)$$

where $\upmu$ $\mathrm{and}\upsigma$ are the mean and the standard deviation of the 20 channels standard deviations respectively, $\mathrm{and}$ ${\widehat{\upsigma }}_{i}$ is the standard deviation of the signal in the $i-th$ channel. If the standard deviation in a channel is greater than Th, the channel is considered noisy and discarded. The discarded channel is either linearly interpolated using the neighbors, or the adjacent channel is used when at the profile edge. This procedure is similar to the one adopted for tomography purposes, where the common practice involves interpolating bad channels using neighbouring ones.

**Fig. 1:**
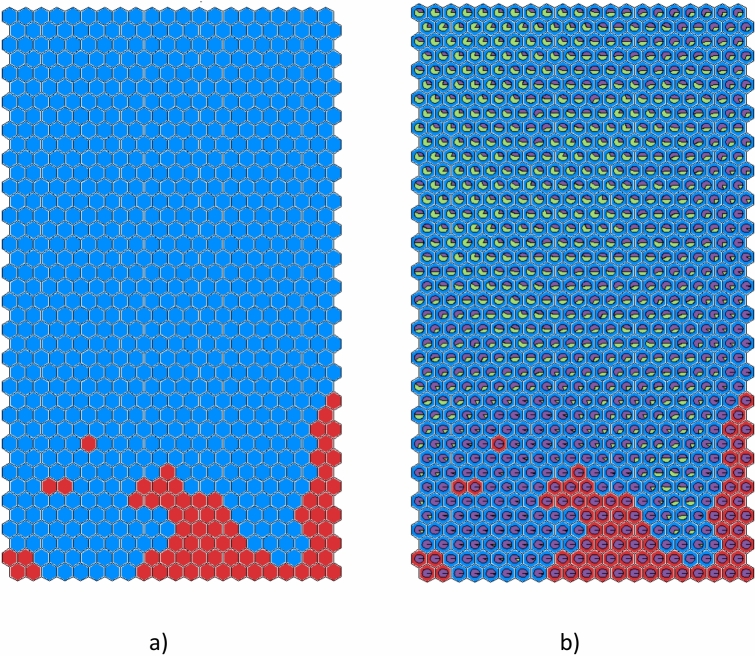
SOM: (**a**) red clusters contain only samples from DTEs; blue clusters may contain samples from DTEs and RTEs or only samples from RTEs; (**b**) the pie planes in each cluster represent the percentage of RTEs (green) and DTEs (purple).

The new SOM, composed of 40 × 21 neurons, is shown in Fig. 1(a). Frontier clusters are labelled as safe, while novel samples are ignored^15^. The red clusters consist exclusively of samples from DTEs. Most clusters contain only samples from RTEs or from both RTEs and DTEs and are blue.

In Fig. 1(b), the pie planes represent the percentage of RTEs (green) and DTEs (purple) in each cluster. As in SOM2^15^, the bottom-left part of the map mainly consists of shots from DTEs even if it is blue due to the presence of samples from RTEs.

**Fig. 2:**
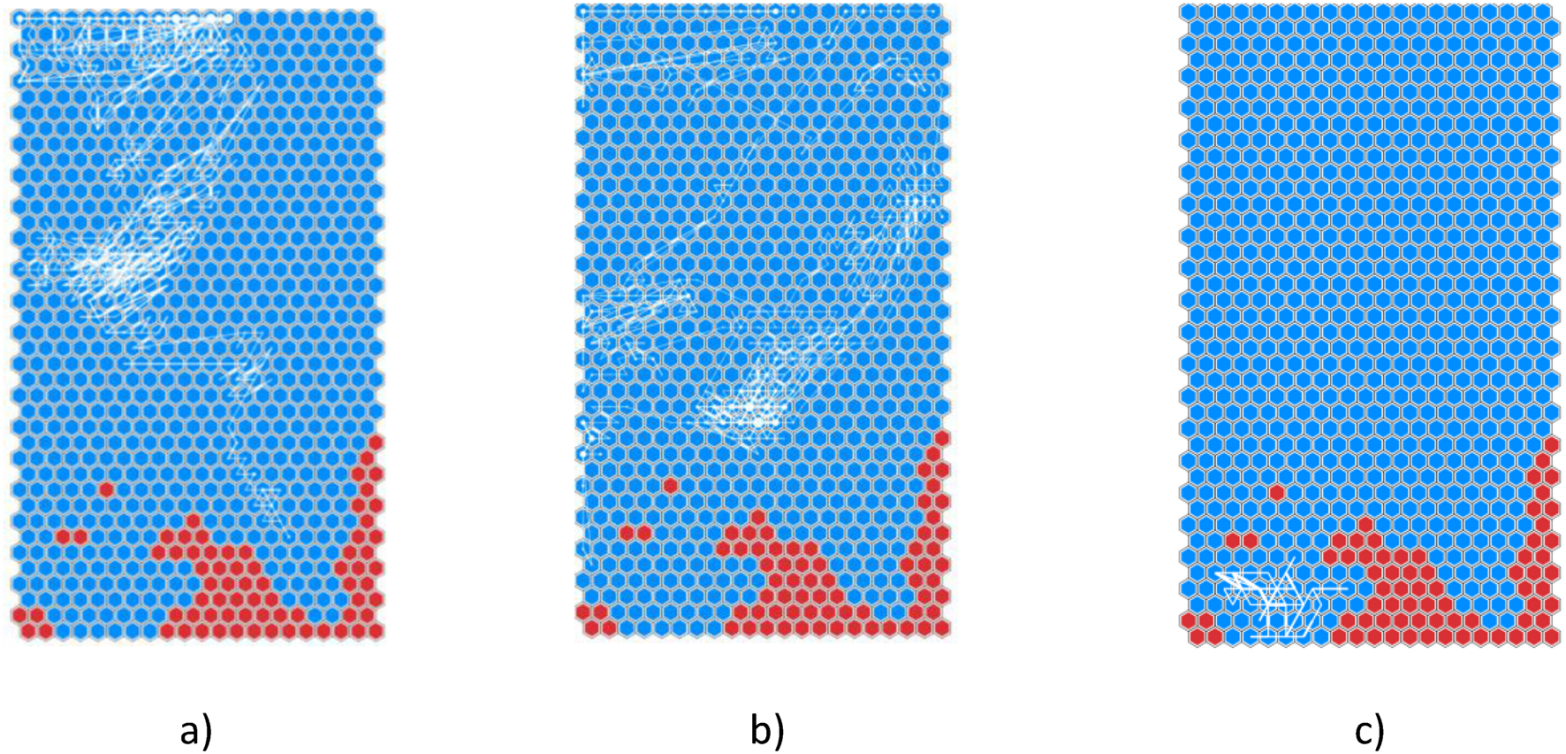
Examples of RTEs trajectories on SOM2: (**a**) #82,194 (**b**) #82,003 (**c**) #82,878 (in white).

Projecting the training RTEs trajectories on the SOM, the majority of RTEs lie far from the red region (Fig. 2). Examples of RTEs trajectories on SOM2: (a) #82,194 (b) #82,003 (c) #82,878 (in white).

(b)) and some of them graze it (Fig. 2).

**Fig. 3:**
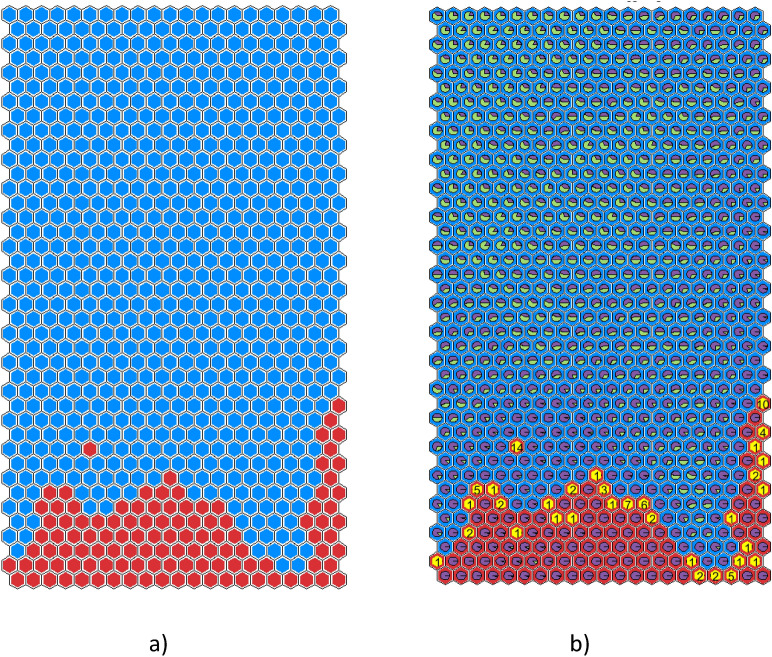
SOM3 relabelled discarding RTE #82,728: (**a**) red clusters contain only samples from DTEs, blue clusters contain samples from RTEs and DTEs or only samples from RTEs; (**b**) the pie planes represent the percentage of RTEs (green) and DTEs (purple) in every cluster.

Only one RTE (#82,728) lies completely in the bottom-left region, as shown by its trajectory traced in Fig. 2.

(c). Its behaviour is compatible with an instability due to impurity accumulation, as confirmed by the comments reported by the operators which observed a W accumulation event and the subsequent cessation of MHD activity. Then, the labels of the SOM clusters are reassigned by discarding RTE #82,728. The resulting map, called SOM3 to distinguish it from SOM2^15^, is shown in Fig. 3(a). A significant change is observed in the subdivision of the map’s regions, now featuring a compact disrupted area that occupies the lower region.

In Fig. 3(b) the pie planes represent the percentage of RTEs (green) and DTEs (purple) belonging to every cluster. Moreover, the SOM clusters where the alarm is triggered are yellow; the number of alarms triggered in the cluster is also reported. The presence of an isolated red cluster where 14 alarms are triggered will be clarified in the following (paragraph 6).

The first two rows of Table 1 report the comparison of the performance between SOM3 and the CNN disruption predictor proposed by the authors^24^ for the same dataset.

**Table 1:** Performance of the different models (CNN^24^, SOM3 and DTr) as disruption predictors.

	Model	FA (%)	MA (%)	TD (%)
Training set	CNN	3 (4.3)	0	0
	SOM3	1 (1.2)	0	0
	DTr	2 (2.4)	0	0
Test set	CNN	14 (9.4)	4 (3.7)	2 (1.9)
	SOM3	6 (4.0)	0	1 (1.0)
	DTr	4 (2.7)	2 (1.9)	1 (1.0)

**Fig. 4:**
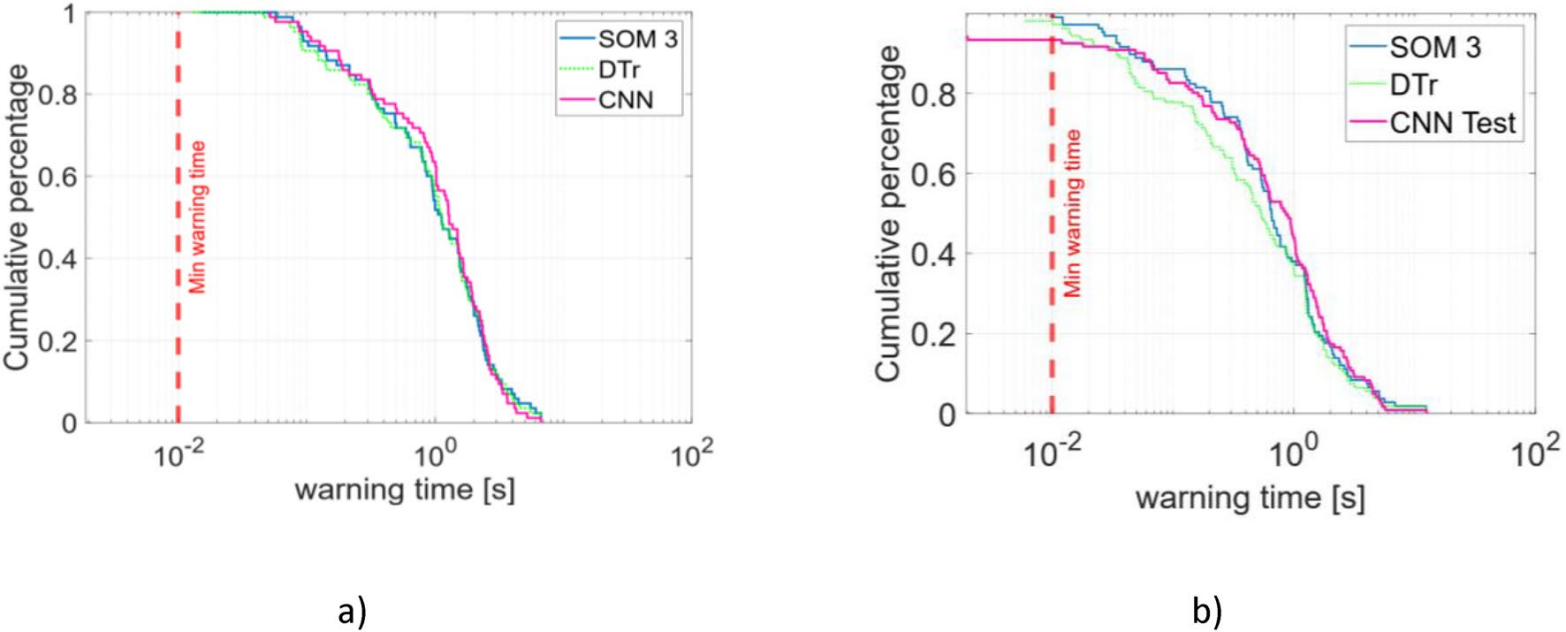
Cumulative fraction of detected disruptions for SOM3 (blue), CNN (magenta) and DTr (green dotted) (**a**) training set (**b**) test set.

The FA in the training set triggered by SOM3 is caused by RTE #82,728 whose trajectory is now in a region labelled as disruptive. The same experiment determines one of the three FAs given by CNN. In Fig. 4 SOM3 and CNN warning times are reported for training (Fig. 4 (a)) and test sets (Fig. 4(b)).

## Rule extraction through decision trees

This work represents a first attempt to explain the boundaries between safe and disruptive regions found by SOM, through the rules extracted by the DTr. The DTr interprets the mechanism behind the SOM answer and identifies disruption root causes triggering the SOM disruption alarm.

The DTr takes as input the same plasma parameters used to train the SOM and as target the corresponding labels as supplied by the SOM. The dataset used for DTr training is the same used to train the SOM. The only difference between them is that, during the training of the tree, the dataset is not under sampled. Moreover, RTE #82,728 is discarded, as result of the new labelling of SOM3.

To ensure model interpretability, the training of the DTr is intentionally limited to two splits. This results in the partitioning of the plasma parameter space into four regions, each represented by a leaf node. Every leaf corresponds to a rule that can be extracted from the tree. With this structure, the model effectively distinguishes between samples from the pre-disruptive phase of DTEs and those from RTEs, replicating the classification capability of the Self-Organizing Map (SOM). The tree accuracy, evaluated as the percentage of samples for which the obtained classification (safe or disrupted) agrees with SOM3 classification, is very high: it is equal to 98.72% for the training set and 98.88% for the test set. As detailed below, this small discrepancy arises from samples located in the clusters at the boundary between the safe and disruptive regions.

**Fig. 5:**
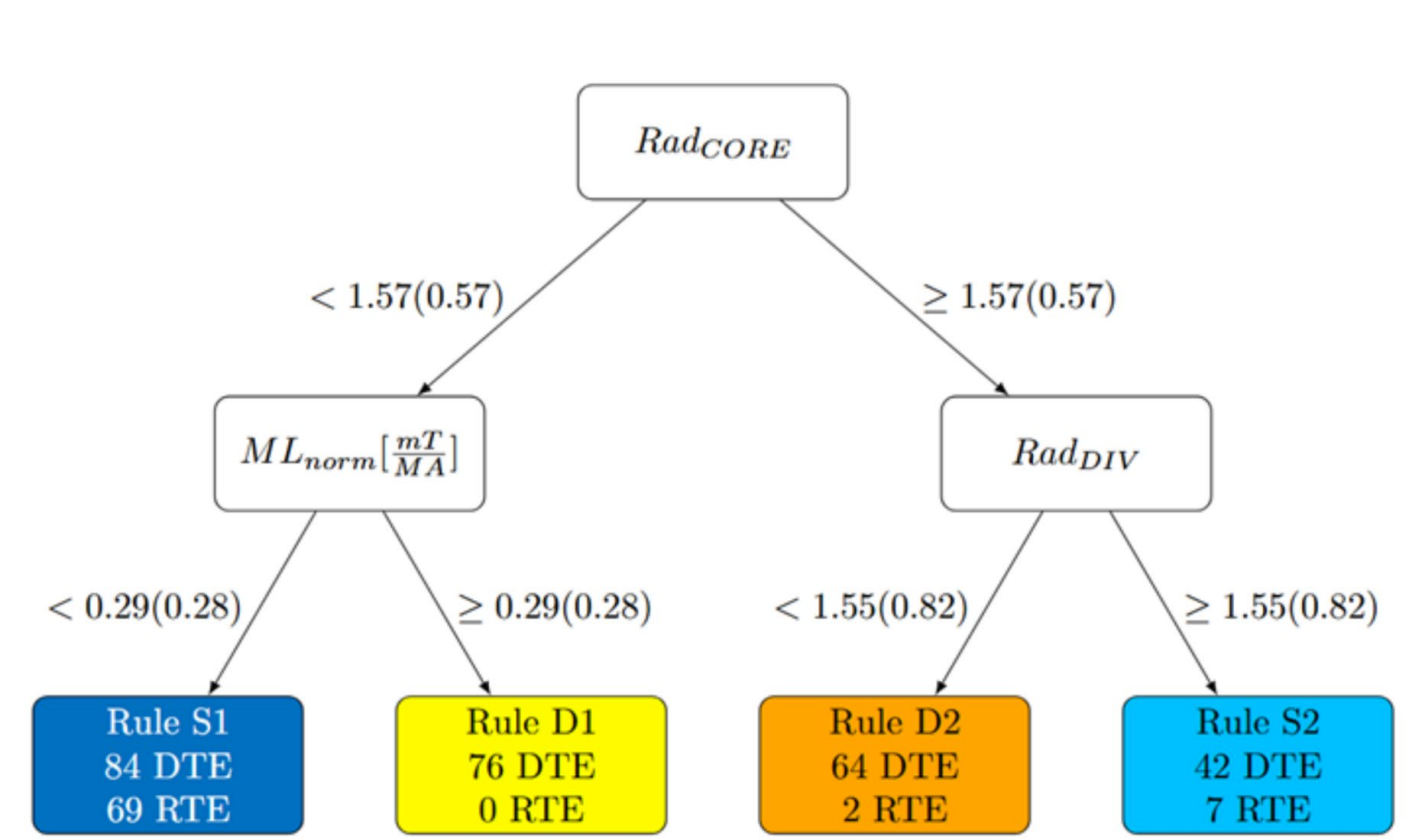
Scheme of DTr; the disrupted leaves are coloured in warm colours (yellow, orange) and the safe ones in cold colours (blue, light blue). In bracket the threshold for normalized signals.

In Fig. 5, the DTr structure is reported, with the number of RTEs and DTEs belonging to the training set associated to each leaf node. Every sample of the training set, associated with one SOM cluster and with the corresponding label, is now associated also with one of the four DTr rules, each one with its color.

The leaves are labelled as Safe and Disrupted depending on the number of DTEs and RTEs samples in the leaf itself. The disrupted leaves are coloured in warm colours (yellow, orange) whereas the safe ones in cold colours (blue, light blue). Note that the presence of leaves where DTEs and RTEs coexist is mandatory since both the experiments start in the same region (i.e., in the region described by rule S1).

By applying the DTr as a disruption predictor, two distinct rules are identified to describe the regions where a disruption alarm is triggered: one associated with the radiation profile destabilization and the other with mode locking:

- Rule D1 yellow: $RA{D}_{pf-CVA}<1.57$ and $M{L}_{norm}\ge 0.29 \left[mT/MA\right]$

This rule is satisfied by 76 DTEs at some point along their trajectory. For 63 DTEs, the DTr firstly triggers the alarm under the D2 rule; then, the label changes to D1 rule in the last part of the experiment. In 23 cases (26.7% of the 85 DTEs) the alarm is triggered for the first time under D1 rule.

- Rule D2 orange: $RA{D}_{pf-CVA}\ge 1.57$ and $RA{D}_{pf-XDIV} < 1.55$

This rule is followed by 64 DTEs and 2 RTEs in some portion of their trajectory. For 63 DTEs (73.3 % of the 85 DTEs) the alarm is triggered for the first time when this condition is satisfied, while for one DTE, the alarm is triggered by rule D1.

This rule identifies disruption precursors related to the destabilization of the radiation profiles exploiting information from radiation peaking factors. In fact, several studies link the radiation profiles to the impurity accumulation and edge cooling mechanism^3,20,23,25,26^ Of the two RTEs that trigger a false alarm, RTE #827283 is the one that was discarded during the training but used as test experiment. Its trajectory follows Rule D2 for most of its samples. Although classified as a FA, this outcome reflects the presence of a destabilizing event due to an impurity accumulation.

Moreover, also the trajectory of the other RTE, #82,447, complies with this rule, for 210 $ms$. Conversely, no alarm is triggered by the SOM for this experiment. In fact, the trajectory passes near to the disrupted region, but it does not enter any disrupted cluster. Again, the presence of high core radiation may be indicative of a destabilizing mechanism affecting plasma performance.

The remaining two rules describe safe regions of plasma parameter space.

- Rule S1: $$RA{D}_{pf-CVA}<1.57$$ and $$M{L}_{norm}<0.29 [mT/MA]$$

It is associated with the majority of samples; all the DTEs and RTEs experiments but two DTEs start in this region, characterized by low values of core radiation and LM. These two DTEs start in disruptive regions, evolving only in the bottom right part of the map.

- Rule S2: $$RA{D}_{pf-CVA}\ge 1.57$$ and $$RA{D}_{pf-XDIV} \ge 1.55$$

This rule is followed by 42 DTEs in some portion of their trajectory and by 7 RTEs.

Summarizing, the DTr splits plasma states into two main branches, the left one depends on the LM for low values of core radiation while the right one only depends on the peaking factors of radiation at the X-point/divertor region for higher values of core radiation.

To evaluate how well the DTr matches the SOM’s clustering results, the performance of the DTr in disruption prediction is reported in 1. Only minor differences are observed in the classification of experiments. Overall, the performance remains comparable, confirming the strong correlation between the extracted rules and the SOM behaviour. The warning times distribution is reported in Figure 4.

## Integrating SOM and decision tree for rule extraction

**Fig. 6:**
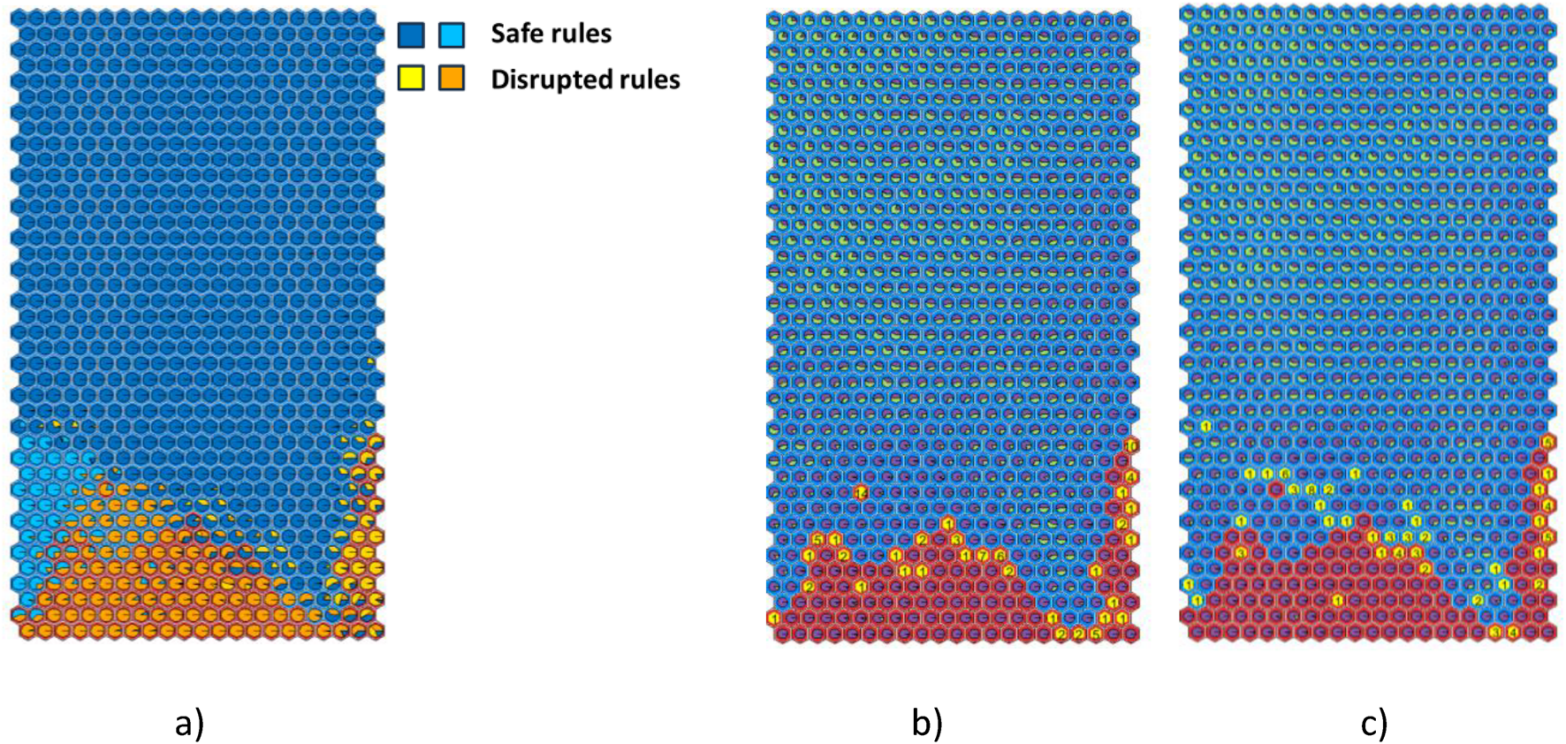
SOM3 (**a**) cluster background depends on the SOM labelling: red for disrupted clusters and blue for the safe ones; the pie planes represent the percentage of samples following a given rule extracted by the DTr (**b**-**c**) the pie planes represent the percentage of RTE samples (green) and DTE samples (purple) in every cluster; the clusters where the alarm is triggered are in yellow with the number of given alarms (**b**) SOM3 alarms (**c**) DTr alarms.

Figure 6(a) shows SOM3 with a cluster background depending on the cluster label: the background is blue for safe clusters and red for disrupted ones. The pie planes in each cluster represent the percentage of samples following a given rule, and they are coloured following the association between colour and rule reported in Fig. 5. In this way, the distribution of the rules is displayed on the SOM.

The boundaries between safe and disrupted regions are the regions where the uncertainty is greater; in fact, as expected, more than one rule is present in the boundary clusters, i.e., rule S1 (blue) coexist in the same cluster with rule D1 (yellow) or rule D2 (orange) on the bottom right side, and rule S1 (blue) with rule S2 (light blue) on the bottom left side. The inner regions, on the other hand, have in general more purity. The presence of some internal orange/light blue clusters is due to the definition of the boundaries in SOM3 itself. The SOM inevitably makes some classification errors due to the nature of its clustering algorithm, which assigns samples to a given cluster based on distances within the multidimensional plasma parameter space.

Moreover, there is a small region where DTr and SOM3 are not in agreement; this is the frontier between the safe and the disruptive one, where the background is blue (safe for SOM3), but the pie-planes show a majority of D2 rule (in orange, disrupted for DTr).

This is more evident in Fig. 6(b) and (c), where the clusters in which the alarm is triggered are yellow together with the number of alarms given by SOM3 (Fig. 6(b)) and by DTr (Fig. 6(c)). The correspondence between DTr and SOM3 alarms mainly differs for the trigger of many DTr alarms in a region considered as safe by the SOM. This is the same frontier region observed in Fig. 6(a), where the background is blue (safe for SOM3) but the pie-planes show a majority of plasma states described by rule D1 (disruptive rule for DTr).

**Fig. 7:**
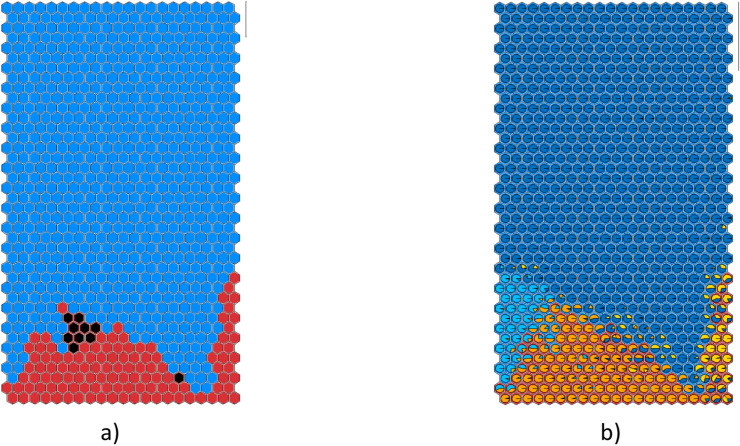
SOM4: (**a**) Red disruptive region and blue safe region after the relabelling following DTr rules; the black dots identify the new disruptive clusters (**b**) the pie planes represent the percentage of samples following a given rule extracted from DTr.

Then, an adjustment of SOM3 labelling is proposed, considering the DTr results: if all the samples in a cluster are classified as disrupted by DTr, then the cluster is labeled as disrupted. The new map, called SOM4, is the combination of the two methods of labelling and is reported in Fig. 7. The red disrupted zone (Fig. 7(a)) is now more widespread. The black dots identify the new disruptive clusters, located almost completely in the area where D2 is the prevalent rule (Fig. 7(b)). The SOM3 cluster triggering 14 alarms is no longer isolated, but it is now at the edge of the disruptive region.

**Fig. 8:**
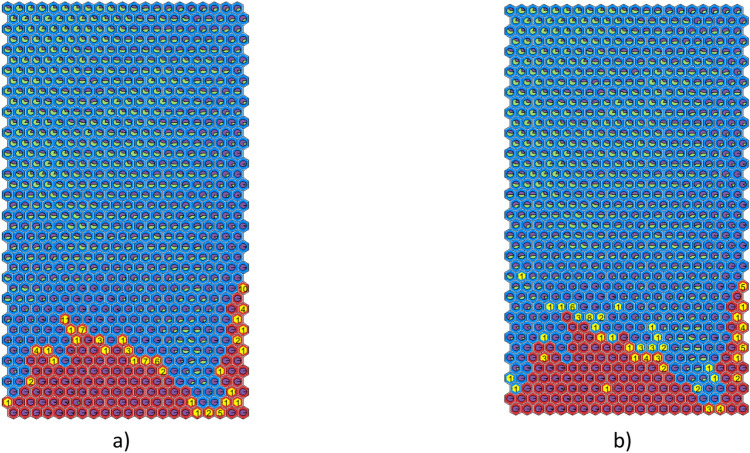
Alarm-triggering clusters are yellow, with the number of alarms (**a**) SOM4 and (**b**) DTr.

In Fig. 8, the clusters in which SOM4 and DTr trigger the first alarm are in yellow with the number of alarms, along with the cluster composition. It can be noticed the better agreement between SOM4 and DTr in terms of region of triggered alarms.

## Interpretation of the DTr rules

The main goal of this work is to propose a procedure for extracting explicit rules based on plasma parameters, providing insight into the decision-making process of a self-organizing map disruption predictor. The resulting set of rules can be interpreted as a series of conditional IF…THEN statements, with each rule corresponding to a leaf in the DTr.

**Fig. 9:**
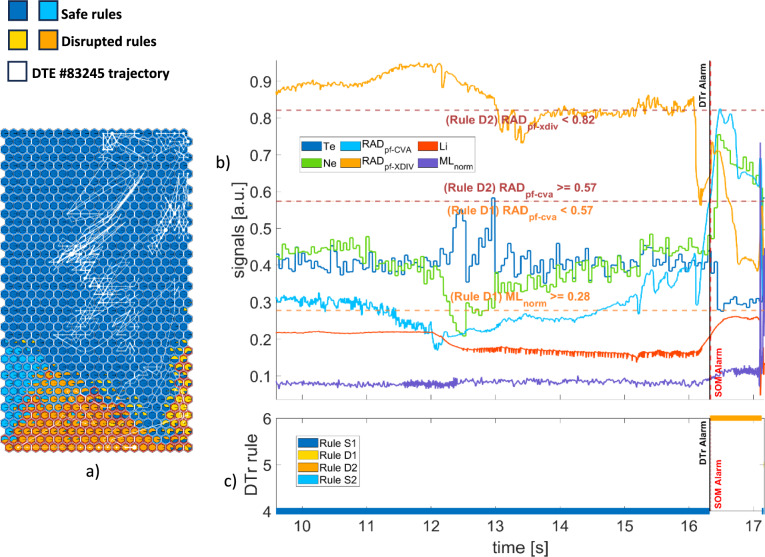
DTE #83,245 – (**a**) SOM trajectory (**b**) plasma parameters and rule thresholds (**c**) rules satisfied by the plasma states.

Here some examples are given to better explain the accordance between the proposed method and other literature works. Figure 9(a) shows the trajectories of DTE #83,245 on SOM3, linked to a core radiation mechanism. The first alarm is given by rule D2. Figure 9(b) shows the time evolution of plasma parameters. Note that the signals and the relative thresholds are normalized in the [0,1] range to enable plotting all signals on a common scale. The DTr alarm and the SOM3 alarm are indicated by vertical lines, black and red respectively (superimposed in the figure). The horizontal dotted lines represent the thresholds characterizing the switching from one rule to another.

In Fig. 9(c), the rules associated with the plasma states are shown in different colours. The experiment starts in the region described by the blue safe rule S1 where both the peaking factor of the radiated power at the plasma core and the LM are under their relative thresholds. Both models classify the samples in this phase as non-disruptive. Then, the divertor radiation starts to decrease and at t = 16.324 s, there is a transition to the orange disruptive rule D2 as soon as the radiated power at core exceeds its thresholds. DTr triggers the alarm; SOM3 still labels the plasma state as safe until t = 16.338 when it also triggers an alarm. The time difference between the two alarms is equal to 14 ms.

**Fig. 10:**
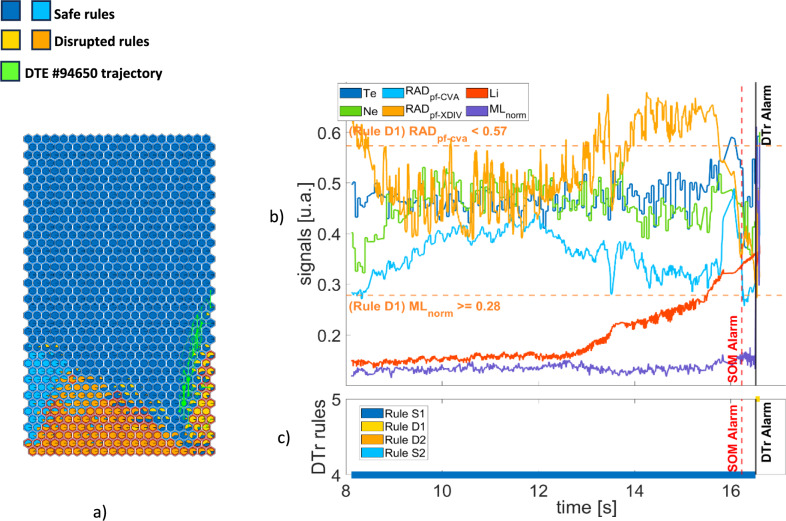
DTE #94,650: (**a**) SOM trajectory (**b**) plasma parameters and rule thresholds (**c**) rules satisfied by the plasma states.

Figure 10(a) shows the trajectory of DTE #94,650 on SOM3. The disruptive mechanism is linked to the LM, and the first alarm is given by rule D1. In this case SOM3 triggers the alarm in advance with respect to the DTr. Figure 10(b) reports the time evolution of the signals: both the peaking factor of the radiated power at the plasma core and the LM are under their relative thresholds. Figure 10(c) reports the rules associated with the plasma states: the experiment is initially classified as safe by both methods (Rule S1). SOM3 triggers the alarm at t = 16.228 s while the DTr switches to the disruptive yellow rule D1 at t = 16.5162 as soon as the LM exceeds the thresholds. The time difference between the two alarms is 0.2846 s.

**Fig. 11:**
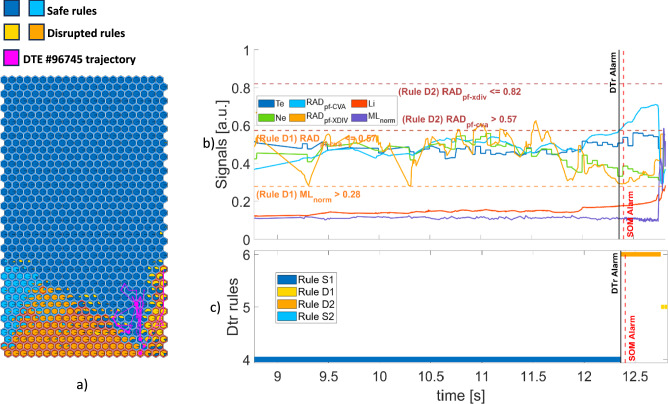
DTE #96,745: (**a**) SOM trajectory (**b**) time evolution of plasma parameters and rule thresholds (**c**) rules satisfied by the plasma states.

Figure 11(a) shows the trajectories of DTE #96,745, linked to an edge radiation mechanism. The first alarm is given by rule D2. The evolution of the plasma parameters is shown in Fig. 11(b). Observing the electron temperature and density profiles from the HRTS, the horizontal bolometer data, the disruption is preceded by an edge radiation collapse at around 12.7s^26^ (Fig. 2, left, in Bonalumi et al.^26^). In Fig. 11(c) the rules associated to the plasma states show that the experiment starts in the region described by the blue safe rule S1 where both the peaking factor of the radiated power at the plasma core and the LM are under their relative thresholds. Both models classify the samples in this phase as non-disruptive. At t = 12.359 s there is a transition to the orange disruptive rule D2 as soon as the radiated power at core exceeds its threshold triggering the DTr rule. Then, SOM3 raises the alarm shortly after, at t = 12.404 s, with a delay of 45 ms. The transition to the D2 rule is 341 ms earlier than the time of the edge radiation mechanism. Finally, the core radiation decreases, and the LM signal exceeds the threshold for the rule D1, causing the DTr to label the final part of the pulse as D1.

**Fig. 12:**
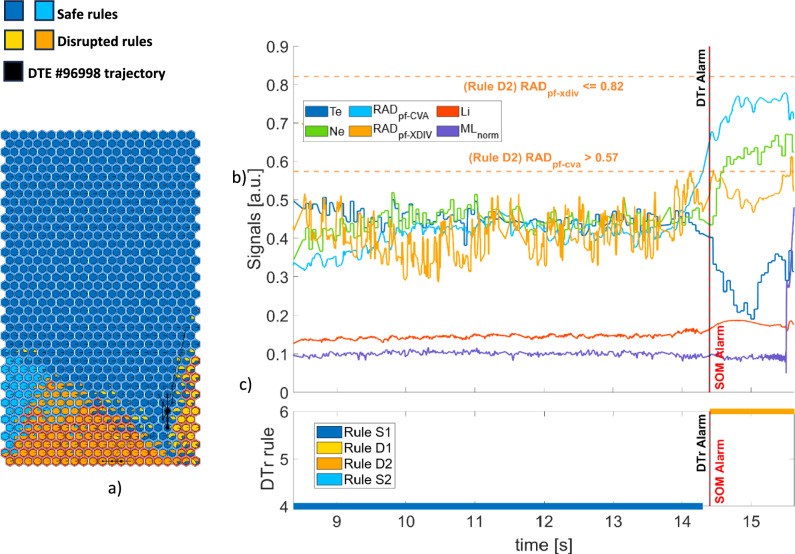
DTE #96,998: (**a**) SOM trajectory (**b**) time evolution of plasma parameters and rule thresholds (**c**) rules satisfied by the plasma states.

Finally, Fig. 12(a) shows the trajectory of DTE #96,998, which is associated with a temperature hollowing disruptive mechanism^26^, with the core cooling down at around 14s (Fig. 2, right, in Bonalumi et al.^26^). Also in this case, the plasma signals in Fig. 12(b) are mapped in the region of the map described by rule S1. Then, at 14.399s, SOM3 triggers an alarm, and the DTr switches the label to the D2 rule, as the $Ra{d}_{pf-CVA}$ overcomes its normalized threshold of 0.57. Some samples before 14.399s are not labelled by any rule since they exceed more than 10% the range of the closest BMU. The transition to the D2 rule is shown in Fig. 12c and is 399ms after the time of the temperature hollowing.

## 3D plasma parameter space

The combination of the SOM’s ability to identify disruptive regions with the DTr’s ability to extract explicit rules enables the visualization of both the plasma parameter space and the disruption prediction rules in a 3D space.

**Fig. 13:**
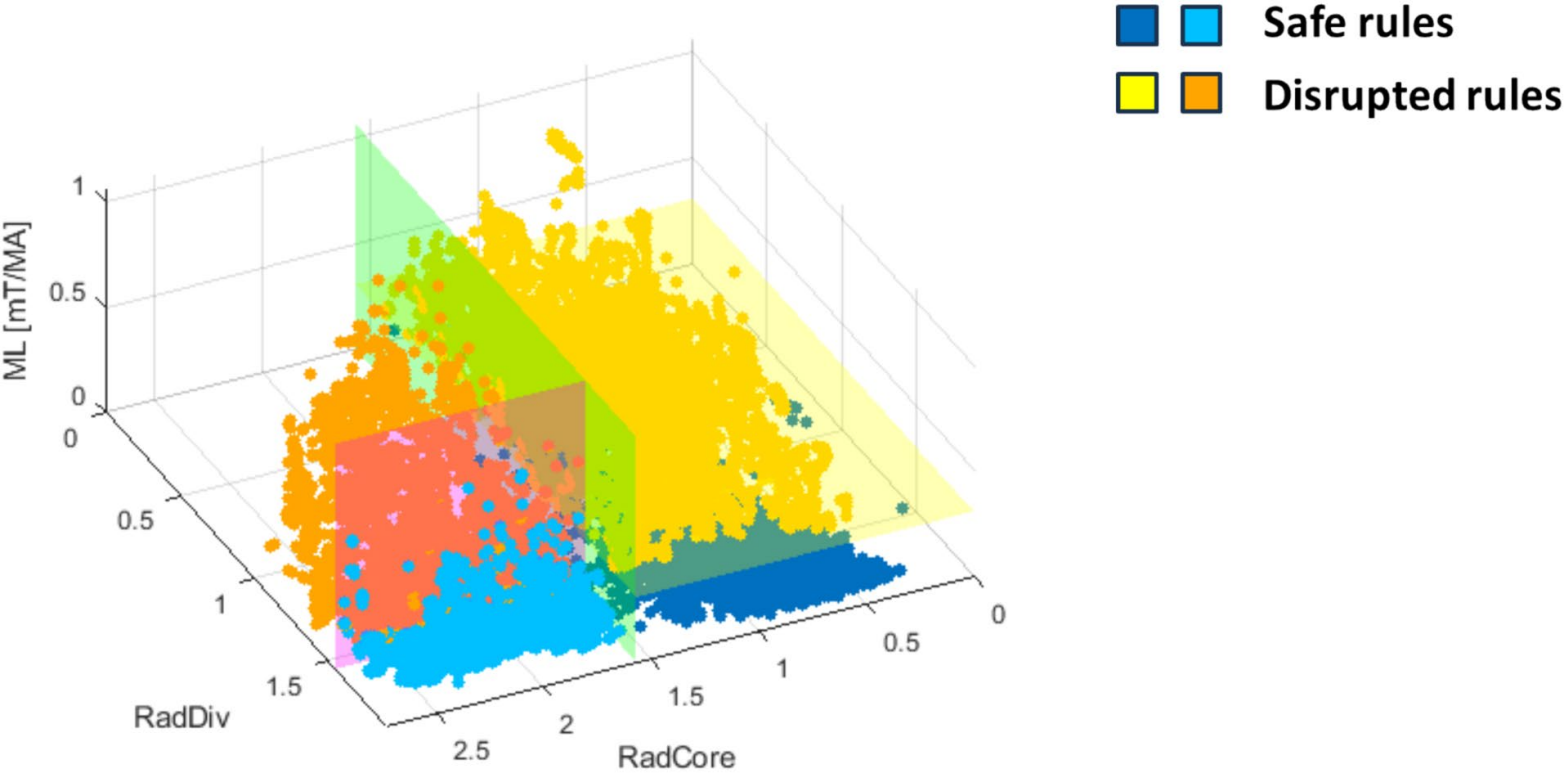
3D plasma states coloured in accordance with the corresponding DTr rule; (see Fig. 5).

Figure 13 illustrates the planar boundaries that separate the regions defined by the four rules. The green plane at RadCore = 1.57 represents the split based on the core radiation peaking factor; the magenta plane at RadDiv = 1.55 corresponds to the split on the edge radiation peaking factor; the yellow plane at ML = 0.29 [mT/MA] marks the split associated with the LM. In the same 3D space, the plasma states can be represented and classified.

A subset of plasma states from the training set is shown here, coloured in accordance with the rules defined by the DTr. The DTr solves multiple binary classification problems by partitioning this 3D plasma parameter space into four regions. This is achieved by establishing decision boundaries, which can be thought of as hyperplanes aligned with the plasma parameter axes. Each plasma state is represented within the 3D space and automatically classified under a specific rule based on its position relative to these boundaries.

## Conclusions

In this work, a first attempt to explain the results of an automatic disruption predictor developed for JET is presented. The predictor is a Self-Organizing Map, trained without any a priori information on the length of the pre-disruptive phase in DTEs. The self-organizing property of SOMs has been exploited to automatically define the regions with high risk of impending disruptions and assign a label (safe/disrupted) to each cluster and consequently to each plasma state in the database, as proposed previously^15^.

The SOM, updated modifying the calculation of the bolometer peaking factors, shows good results both in terms of false alarms and missed alarms.

Moreover, the labelled database achieved by the Self-Organizing Map has been used to train a Decision Tree, to extract a set of rules able to adapt and describe the different boundaries between safe and disruptive regions on the Self-Organising Map. Then, the integration of Self-Organising Map and Decision Tree outcomes allowed one to review the boundaries of safe and disruptive regions firstly identified by the Self-Organising Map.

The results obtained show the agreement between Self-Organising Map and Decision Tree. In fact, predicting disruptions using the rule set provides results close to those shown by the Self-Organising Map.

The synergy between these two methods lies in their complementary roles: the Self-Organising Map performs unsupervised clustering capturing complex patterns in data, while the Decision Tree extracts interpretable rules that explain how the Self-Organising Map classifies the samples. This combination is highly effective, improving both accuracy and transparency. The integrated model is highly interpretable and enhances the possibility to understand the disruption trigger causes in a straightforward way.

The results are constrained by the limited extent of the database, but the next application of the method on other tokamak data stored in the EUROfusion database and the identification of relevant plasma parameters and scale factors among different machines will bring to a better understanding of disruptions and ultimately to a multi-machine model, determinant to the future ITER experiment.

## Data Availability

The JET experimental data is stored in the PPF (Processed Pulse File) system which is a centralised data storage and retrieval system for data derived from raw measurements within the JET Torus, and from other sources such as simulation programs. These data are fully available for the EUROfusion consortium members and can be accessed by non-members under request to EUROfusion. Numerical data supporting the outcome of this study are available from the corresponding author upon request.
